# Hybrid Eu(II)-bromide scintillators with efficient 5*d*-4*f* bandgap transition for X-ray imaging

**DOI:** 10.1038/s41377-024-01589-w

**Published:** 2024-08-29

**Authors:** Kai Han, Jiance Jin, Yuzhen Wang, Xinquan Zhou, Yongsheng Sun, Lihan Chen, Zhiguo Xia

**Affiliations:** 1 The State Key Laboratory of Luminescent Materials and Devices, Guangdong Provincial Key Laboratory of Fiber Laser Materials and Applied Techniques, Guangdong Engineering Technology Research and Development Centre of Special Optical Fiber Materials and Devices, School of Physics and Optoelectronics, South China University of Technology, Guangzhou, China; 2 School of Materials Science and Engineering, South China University of Technology, Guangzhou, China

**Keywords:** Optical materials and structures, Applied optics

## Abstract

Luminescent metal halides are attracting growing attention as scintillators for X-ray imaging in safety inspection, medical diagnosis, etc. Here we present brand-new hybrid Eu(II)-bromide scintillators, 1D type [Et_4_N]EuBr_3_·MeOH and 0D type [Me_4_N]_6_Eu_5_Br_16_·MeOH, with spin-allowed 5*d*-4*f* bandgap transition emission toward simplified carrier transport during scintillation process. The 1D/0D structures with edge/face -sharing [EuBr_6_]^4−^ octahedra further contribute to lowing bandgaps and enhancing quantum confinement effect, enabling efficient scintillation performance (light yield ~73100 ± 800 Ph MeV^−1^, detect limit ~18.6 nGy s^−1^, X-ray afterglow ~ 1% @ 9.6 μs). We demonstrate the X-ray imaging with 27.3 lp mm^−1^ resolution by embedding Eu(II)-based scintillators into AAO film. Our results create the new family of low-dimensional rare-earth-based halides for scintillation and related optoelectronic applications.

## Introduction

Scintillators, producing visible photons by transforming high-energy X-rays, have been widely applied in scientific research, safety inspection, medical diagnosis, etc^1,2^. In the last decades, solution-processed scintillating materials, e.g., metal-organic frameworks^3,4^, organic molecules^5–7^, metal halide perovskites^2,8,9^, and hybrid metal halides (HMHs)^10–12^, have emerged as promising alternatives to conventional inorganic scintillators due to the characteristics of low-cost and easy-processing. In particular, HMHs with metal-halide polyhedrons isolated by organic cations possess inherent merits in terms of tunable structure determination and luminescence, effective radioluminescence, and heavy-atom (metals and halides) for X-ray absorption^13^. However, most of the present HMH scintillators are difficult to meet requirements for the state-of-the-art X-ray imaging, such as Mn(II)/Cu(I)-based HMHs (long decay time) and Pb(II)-based compounds (environmental toxicity)^14–17^. Thus, the exploration of novel HMH scintillators that are highly sensitive to X-rays is of high importance.

**Scheme 1:**
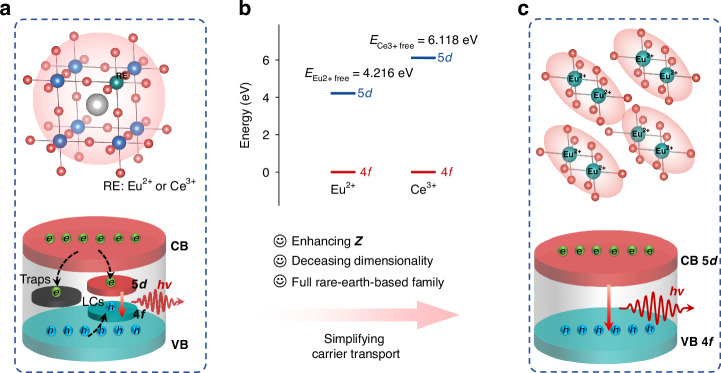
**a** The scintillation process of traditional rare-earth doped scintillators. **b** The energy bandgaps free ions Eu^2+^ and Ce^3+^. **c** Mechanism illustrations and rational design of RE-based scintillators with efficient 5*d*-4*f* bandgap emission

Radioluminescence of scintillators generally undergo three stages^18–20^. First, the large amounts of electron-hole pairs are generated under X-ray radiation interacting with the heavy atoms in scintillators. Second, the charge carriers transport to defects or the bottom of the conduction band (CB) and the top of the valence band (VB), and captured at the luminescence centers (LCs) of scintillators, such as rare-earth Eu^2+^ 4*f*-5*d* (Scheme 1a). Last, radiative recombination of captured carriers at the LCs generates visible radioluminescence. Accordingly, it can be extrapolated that three elements induce efficient radioluminescence, i.e., heavy atoms, few defects and low energy level D-value between CB/VB and LCs, and high luminescent efficiency. These factors are mainly dominated by regulating appropriate B-site ions, e.g., rare-earth ions (Ce^3+^ and Eu^2+^). However, the Eu-based halide scintillators generally possess higher light yield for Ce-based counterparts, such as Cs_4_EuBr_6_: 78000 Ph MeV^−1^, Cs_3_CeBr_6_: 28000 Ph MeV^−1^ ^21,22^. Moreover, the energy bandgaps (*E*_Eu2+ free_ = 4.216 eV) of free ions (Eu^2+^ 5*d*-4*f*) is much smaller than that (*E*_Ce3+ free_ = 6.118 eV) of Ce^3+^ 5*d*-4*f*^23^, which enhances the feasibility of synthesizing Eu^2+^-based small bandgap hybrid materials and obtaining higher light yield, as shown in Scheme 1b. Therefore, we reason the Eu^2+^ ions that can be considered as B-site ones to design brand-new HMH scintillators based on their heavy atom effectivity and spin-allowed 5*d*-4*f* bandgap emission for efficient carrier transport (Scheme 1c). Additionally, reduced molecular dimensionality in HMHs could be employed to enhance luminescent efficiency due to the localized charge carriers (Scheme 1c)^24,25^. From optical imaging perspective, in addition, various large-area HMH scintillators were explored, i.e., nanocrystal thin films, thick films, hybrid film (mixture of HMH and polydimethylsiloxane), and transparent ceramics/glasses^10,11,26^. However, the abovementioned forms remain challenging for durability or poor optical transparency/output. Combining with solution processability of HMHs, their AAO films are thought to generate optical waveguide effect and thus enhance X-ray imaging quality. Despite several Eu^2+^-doped/-based scintillators, such as Cs_4_EuBr_6_, SrI_2_:Eu^2+^, LiI: Eu^2+^ and BaCl_2_:Eu^2+^, have been reported^22,27–29^, the synthesis conditions are challenging, including high-cost, time-consuming, high-temperature, and vacuum/high-pressure sintering processes. Thus, the solution processability of rare-earth-based scintillators combining highly sensitive to X-rays is an alternative way.

Here we demonstrate an example of Eu(II)-based scintillators from adjusting molecular dimensionality and coordination environment in 3D CsEuBr_3_ (corner-shared octahedra) and discovered two low-dimensional Eu(II)-based scintillators (one is 1D type [Et_4_N]EuBr_3_·MeOH (***EtEu***) with edge-shared octahedra; the other is 0D type [Me_4_N]_6_Eu_5_Br_16_·MeOH (***MeEu***) with face-shared octahedra). The scintillation of ***MeEu*** shows great light yield of ~73100 ± 800 Ph MeV^−1^, low detection limit of 18.6 nGy s^−1^, weak X-ray afterglow of ~1% @ 9.6 μs. The efficient scintillation performance is originated from 4*f*-5*d* bandgap emission, lowing bandgaps and enhancing quantum confinement effect. Furthermore, X-ray scintillation imaging by embedding Eu(II)-based scintillators into AAO film exhibits ultrahigh spatial resolution of ca. 27.3 lp mm^−1^. These findings provide valuable guidelines for designing novel HMH scintillators with high sensitivity and low cost.

## Results

### Design and structural characteristics

As a conceptual design, we selected tetramethylammonium bromide (Me_4_NBr) and tetraethylammonium bromide (Et_4_NBr) as organic cations due to good processability. The anhydrous MeOH with high polarity of ca. 6.6 is able to dissolve Me_4_NBr/Et_4_NBr and EuBr_2_ to form a clear solution and safeguard B-site Eu(II) from oxidation^30^, as shown in Fig. S1. The colorless and viridescent single crystals can be easily obtained with dissolvent volatilization and emit bright blue and cyan emission under ultraviolet (UV) irradiation, respectively (Fig. S2). The details of the synthesis process are depicted in the Methods section. Scanning-electron-microscope (SEM) images of as-prepared crystals reveal high crystallization quality, and energy-dispersive-spectrometer (EDS) images with homogenous distribution of N, Eu and Br elements confirm that we synthetize new hybrid Eu(II) halides (Fig. S3). Considering the solution processability of these materials, the large-area scintillation screens can be facilely fabricated enabling X-ray imaging.

**Fig. 1:**
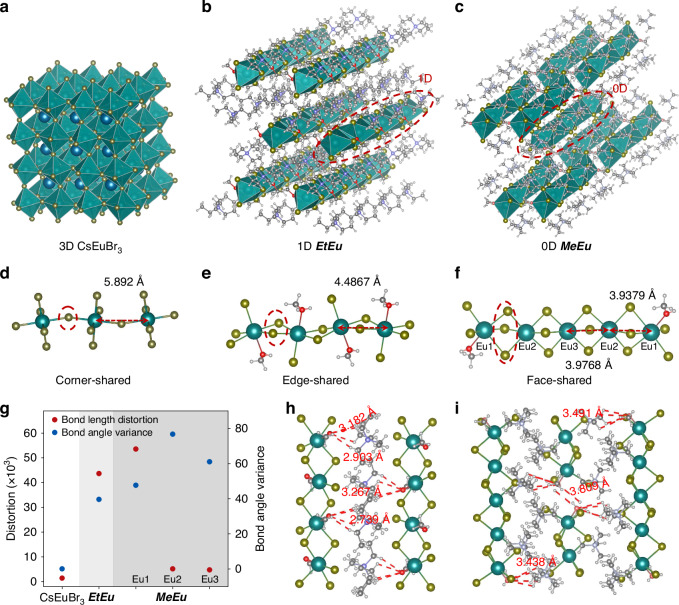
**Structural characteristics of Eu(II)-based hybrids**. Crystal structures of **a** 3D CsEuBr_3_, **b** 1D ***EtEu*** and **c** 0D ***MeEu*** hybrids. Local connection pattern of [EuBr_6_]^4−^ octahedrons with **d** corner-sharing for CsEuBr_3_, **e** edge-sharing for 1D ***EtEu***, and **f** face-sharing for 0D ***MeEu***. **g** Calculated bond length distortion of bond angle variance of the compounds. Highlights of the hydrogen bonds marked in red dashed line between organic cations and MeOH in the **h** 1D ***EtEu*** and **i** 0D ***MeEu*** hybrids

**Fig. 2:**
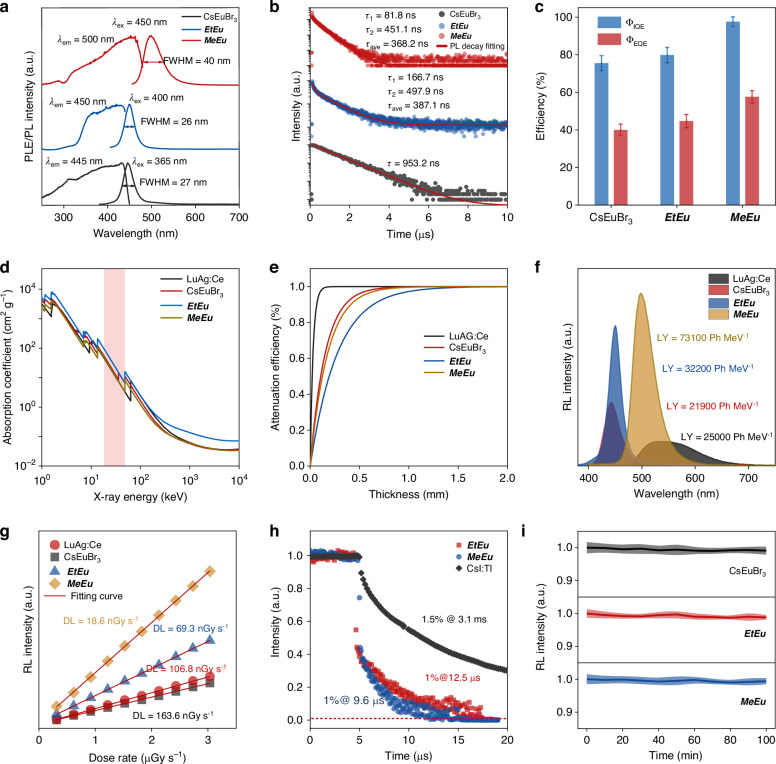
**Photoluminescence/radioluminescence characterizations of Eu(II)-based halides. a** Normalized PL and PLE spectra at room temperature of CsEuBr_3_, ***EtEu*** and ***MeEu*** halides. **b** PL decay of CsEuBr_3_, ***EtEu*** and ***MeEu*** halides as a function of time recorded at the PL maximum peaks under 340 nm pulsed excitation. **c** *Φ*_IQE_ and *Φ*_EQE_ of CsEuBr_3_,***EtEu*** and ***MeEu*** halides, and the *Φ*_EQE_ is equal to the *Φ*_IQE_ multiplied by the absorption coefficient. **d** X-ray absorption spectra of CsEuBr_3_, ***EtEu*** and ***MeEu*** halides and commercial LuAG:Ce^3+^ scintillator. **e** Calculated X-ray attenuation efficiencies of CsEuBr_3_, ***EtEu*** and ***MeEu*** halides and LuAG:Ce^3+^ versus thickness at photon energy of 17.5 keV. **f** RL intensity spectra CsEuBr_3_, ***EtEu*** and ***MeEu*** halides and LuAG:Ce^3+^ with normalized to the X-ray attenuation efficiencies at the thickness of 500 μm (X-ray tube voltage, 50 kV; dose rate, 1.503 mGy s^−1^). **g** Detection limits under low-dose-rate X-ray excitation at SNR of 3. **h** The afterglow of commercial CsI:Tl, ***EtEu*** and ***MeEu*** halides under X-ray off. **i** Normalized RL intensities of 3D CsEuBr_3_, 1D ***EtEu*** and 0D ***MeEu*** halides a total dose of 8.4 Gy during X-ray irradiation

We measured single crystal X-ray diffraction (SCXRD) to gain crystal complementary data. Detailed crystal cell parameters summarized in Tables S1–S3. Compared with 3D structure of CsEuBr_3_ (Fig. 1a), crystal of [Et_4_N]EuBr_3_·MeOH (***EtEu***) posseses one-dimensional (1D) chain structure isolated by Et_4_N^+^ cations (Fig. 1b), whereas [Me_4_N]_6_Eu_5_Br_16_·MeOH (***MeEu***) only contains zero-dimensional (0D) [EuBr_6_]^4−^ polyhedron surrounded by Me_4_N^+^ cations (Fig. 2c). Notably, the strong quantum confinement from reduced structural dimensionality is able to enhance luminescence efficiency^24^. The high phase-purity of ***EtEu*** and ***MeEu*** can be further approved by Powder X-ray diffraction (PXRD) patterns (Fig. S4). The XPS spectrum is employed to demonstrate the testify divalent valence state of Eu ions in ***EtEu*** and ***MeEu*** hybrids (Fig. S5)^31,32^. In particular, coordination environment of Eu(II)-Br transfer from corner-shared to edge-shared octahedra, and even face-shared octahedra with structural dimensionality decreasing from 3D to 0D (Fig. 1d, e). The differences in local coordination could affect bandgap structure^33,34^. The Eu–Eu metallic bonding in face-shared motifs (3.9379–3.9768 Å) is obviously shorter than ones in edge/corner-shared clusters (5.892 Å and 4.4867 Å). The face/edge-shared structures, with stronger couple of [EuBr_6_]^4−^ polyhedrons, exhibits good structural stability and low trap states^34–36^. The distortion levels of [EuBr_6_]^4–^ octahedra are calculated and summarized in Fig. 1g. The face/edge-shared [EuBr_6_]^4–^ octahedra reveals higher distortion level from the bond length distortion and bond angle variance, which is similar to the tendency between distortion level and connectivity modes in the 2D lead bromide perovskites^33^. Meanwhile, we also observe that the MeOH molecules coordinate to Eu in [EuBr_5_·MeOH]^3–^ octahedra, resulting in locally collective hydrogen bonding. By hydrogen bonding comparison, ***EtEu*** process stronger hydrogen bonding effect along the direction perpendicular to the 1D chains, preserving 1D structure (Fig. 1h). However, the chain structure is destroyed by the steric effects of organic cation in ***MeEu*** without muscular hydrogen bonding, forming 0D clusters (Fig. 1i)^37^. The forementioned results indicate that ***EtEu*** and ***MeEu*** hybrids owns quantum confinement, structural stability and decreasing trap states, contributing to enhanced luminescent properties.

### Photoluminescence/radioluminescence properties

Figure 2a displays the steady-state photoluminescence (PL) and excitation (PLE) spectra of 3D CsEuBr_3_, 1D ***EtEu*** and 0D ***MeEu*** hybrids, respectively, with detailed PL parameters summarized in Table S4. Remarkably, under UV excitation from 300 to 400 nm (Fig. S6a), 3D CsEuBr_3_ shows narrow-blue emission peaking at 445 nm with full width at half maximum (FWHM) approximately 26 nm. Similarly, 1D ***EtEu*** also produces narrow-blue emission (FWHM ~ 24 nm) peak at 455 nm when excited by UV from 270 to 400 nm (Fig. S6b), while 0D ***MeEu*** hybrids exhibit narrow-cyan photoluminescence (FWHM ~ 40 nm) centered at 500 nm with broad PLE band ranging from 300 to 445 nm (Fig. S6c). The emission redshift of ***MeEu*** hybrids with higher Stokes shift (Δ*S*(A)) is contributed by higher Huang−Rhys (HR) factor ~6.58 (Fig. S7)^38^. Following Dorenbos’ semiempirical model^39,40^, the *E*_abs_ and *E*_em_ of CsEuBr_3_, ***EtEu*** and ***MeEu*** hybrids were calculated and almost consistent with experimental results (Table S5). Apparently, the emissions of the designed HMHs are originated from the 4*f*–5*d* transitions of Eu(II). The luminescence decays at the emission maximum as bi/mono-exponential functions for 3D, 1D ***EtEu*** and 0D ***MeEu*** with short lifetime of 953.2 ns, 387.1 ns, and 368.2 ns (Fig. 2b), respectively. The decreasing luminescence lifetime is mainly attributed to shorter Eu-Eu distances (*d*_Eu-Eu_ (CsEuBr_3_) ~ 5.892 Å, *d*_Eu-Eu_ (***EtEu***) = 4.4867 Å, *d*_Eu-Eu_ (***MeEu***) ~ 3.9379–3.9768 Å)^41,42^. The typical PLE, PL and PL lifetime spectra indicate their emission is indeed originated from the spin-allowed 4*f*–5*d* transitions of Eu(II)^43^. It should be pointed out that the short decaying lifetime in 1D ***EtEu*** and 0D ***MeEu*** hybrids is great significance for fast X-ray scintillation imaging. Impressively, photoluminescent internal/external quantum efficiency (*Φ*_IQE_ and *Φ*_EQE_) gradually increase from 78%/43% to 97%/61% accompanied with reduced molecular dimensionality (Fig. 2c and Table S6). Moreover, the energy barriers of thermal quenching Δ*E* of CsEuBr_3_, ***EtEu*** and ***MeEu*** hybrids are at the same level as most Eu^2+^ doped inorganic phosphors (Fig. S8), meaning a good thermal stability^44^. As the thermal stability of ***MeEu*** remained above 80% at 100 °C (Fig. S9), thanks to its higher Δ*E* value and higher structural stability (more rigid face-sharing local structure and organic cations isolated 0D structure). Strikingly, the enhanced quantum confinement in low-dimensional hybrid gives rise to the higher *Φ*_IQE_ and *Φ*_EQE_, which are also in favor of efficient scintillation performance.

It is noteworthy that the X-ray absorption coefficients of [EuBr_6_]^4−^ octahedra (*Z* = 35 and *K*_α_ = 13.475 keV for bromine; *Z* = 63 and *K*_α_ = 48.515 keV for europium) is far outweigh than those of organic cations (*Z* = 1–8, *K*_α_ = 0.0136–0.531 keV for C, H, N and O). As a result, 3D, 1D and 0D Eu(II) bromides show identical resonant absorption edges, even commercial scintillator LuAG:Ce (Fig. 2d). To investigate the radioluminescence (RL) properties, we compare X-ray attenuation efficiency *vs* sample thicknesses (Fig. 2e). The difference of X-ray attenuation efficiency is related to the densities of 3D CsEuBr_3_, 1D ***EtEu***, and 0D ***MeEu*** hybrids with 4.31 g cm^−3^, 2.19 g cm^−3^, and 2.51 g cm^−3^, respectively. Moreover, we evaluate light yields (LY) of X-ray to visible photons (Figs. 2f), and 1D ***EtEu*** and 0D ***MeEu*** exhibit high LY of ~ 73100 ± 800 and ~ 32200 ± 700 ph MeV^−1^, all of which evidently outperform those of 3D CsEuBr_3_ (21900 ± 300 ph MeV^−1^) and LuAG:Ce (25000 ph MeV^−1^), related to their high luminescent efficiencies and low optical bandgaps. The low detection limit of 18.6 nGy s^−1^ in 0D ***MeEu*** (Fig. 2g), <0.5% of X-ray diagnostic dosage of 5.5 μGy s^−1^ ^45^, is inferred by linear dependence of RL intensity on X-ray dose rate at signal-to-noise ratio (SNR) ~ 3 (Fig. S10). As-prepared Eu(II) scintillators reveal superiority in X-ray afterglow respect. As shown in Fig. 2h, the afterglow of 1D ***EtEu*** and 0D ***MeEu*** is 1% @12.5, 9.6 μs, respectively, which is superior to the commercial scintillator CsI:Tl (1.5% @ 3.1 ms)^46,47^. In addition, the X-ray fatigue stability is another important limiting factor of metal halide scintillators. Figure 2i illustrates the RL intensity of Eu(II)-based halides exposure at a total dose of 8.4 Gy, demonstrating the superb stability. Consequently, it can be concluded that the 0D ***MeEu*** process transcendent X-ray scintillation performance, propelling the favorable for X-ray imaging devices by solution processed scintillation screens.

### Modified radioluminescence mechanism

**Fig. 3:**
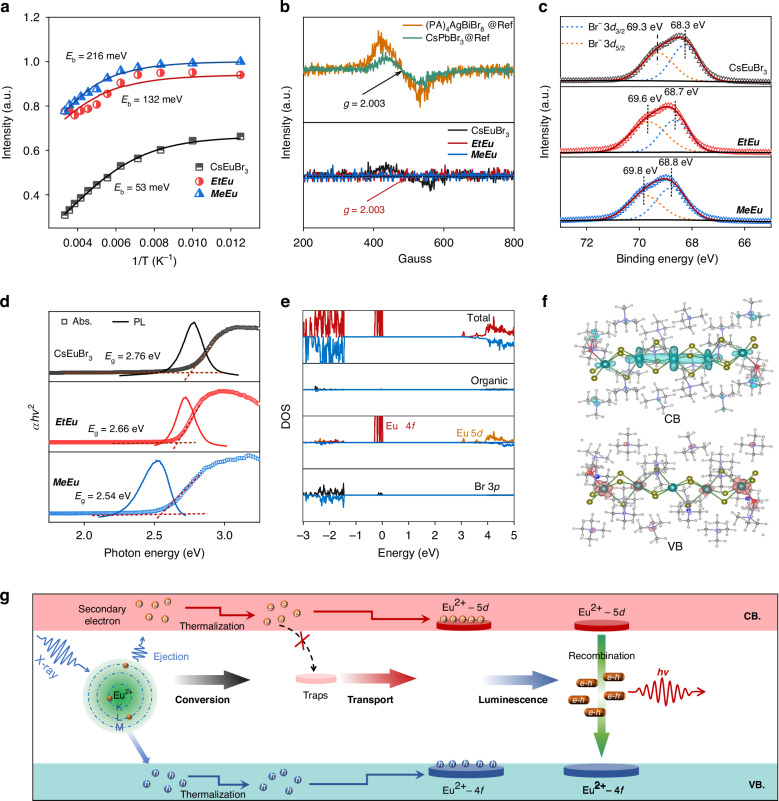
**Mechanism of scintillation in Eu-based halides. a** Fitting results of the integrated PL intensity–temperature dependence of CsEuBr_3_, ***EtEu*** and ***MeEu*** halides. **b** EPR spectra, and **c** XPS spectra Br-3*d* core level of CsEuBr_3_, ***EtEu*** and ***MeEu*** halides. **d** The plots of (*αhv*)^2^ and PL versus the light energy of CsEuBr_3_, ***EtEu*** and ***MeEu*** hybrids. **e** The density of states (DOS) plots of ***MeEu***. **f** Partial density contours in the VB and CB for ***MeEu***. **g** Proposed mechanism of radioluminescence for Eu-based halides

Generally, the scintillation performance is determined by bandgap (*E*_g_) of the scintillator, and quantum efficiencies in the transfer (*S*) and luminescence stages (*Q*) as follows^48^:

1 $${\rm{LY}}=\left({10}^{6}/\beta {E}_{g}\right){SQ}$$

where *β* is a phenomenological parameter (2 ~ 3). With the goal of revealing origination of efficient luminescence, we first turned to studying the exciton binding energy *E*_b_ by Arrhenius fitting from temperature-dependent PL (Fig. S11)^49^.

2 $$I(T)=\frac{{I}_{0}}{1+A\exp \left(-\frac{{E}_{b}}{{k}_{B}T}\right)}$$

where *I*_0_ is the intensity at 0 K and *k*_B_ is the Boltzmann constant. It was found that ***MeEu*** halide displayed a larger value (216 meV) of the *E*_b_ than those (132 meV and 53 meV) of other two halides (Fig. 3a), manifesting the effect of quantum confinement in effectively enhancing luminescence. This trend matches well with their corresponding scintillation performance. Typically, the defect states in metal halides originate from the vacancies of halide. Thus, we used electron paramagnetic resonance (EPR) measurement (Fig. 3b) to estimate the concentration of Br vacancy (*V*_Br_)^50,51^. The three samples show lower a characteristic signal for *g*-factor of 2.003 than that of (PA)_4_AgBiBr_8_ and CsPbBr_3_, reflecting lesser defect states for as-prepared Eu-based halides. The lower defect states (*V*_Br_) could be further corroborated by shifting higher binding energy of Br 3*d*_5/2_ and 3*d*_3/2_ in Eu-based halides (Fig. 3c)^52,53^, and no spectral signal at thermoluminescence measurement (Fig. S12)^54^. The lower The defect states contribute to short X-ray afterglow performance^55^. This observation could be attributed to the more rigid local structure (face-sharing > edge-sharing > corner-sharing).

The optical bandgaps were calculated from UV-vis diffuse reflectance spectra (Fig. 3d) with the values of 2.77 eV for CsEuBr_3_, 2.66 eV for ***EtEu***, 2.54 eV for ***MeEu***, indicating improved probability of captured electron-hole pairs carriers under X-ray radiation^19^. Additionally, we also observed good matching at energy between absorption edge and PL emission at room temperature (Fig. 3d), which describes band-to-band (5*d*-4*f*) recombination in these Eu(II)-based halides^56^. To gain further insight into luminescent mechanism of 3D CsEuBr_3_, 1D ***EtEu*** and 0D ***MeEu***, we performed density functional theory (DFT) calculations on the electronic configurations. The direct bandgap can be observed in all systems with ca. 3.39 for 3D CsEuBr_3_, 3.10 for 1D ***EtEu*** and 2.95 eV for 0D ***MeEu*** (Fig. S13), which is consistent with results of absorption data. The dispersive bands and smaller band gap in 1D edge/0D face-shared structures in comparation with 3D corner-ones clearly illustrates that the molecular dimensionality and connectivity of the octahedra is impacted on the electronic structure^33,34^. The projected density of states (DOS) shows that valence band (VB) is mainly contributed by the 4 *f* orbitals of Eu(II), whereas the conductive band (CB) is derived from 5*d* orbitals of Eu(II) (Fig. 3e and Fig. S14). There is no contribution from the Br 4*p* or organic cations to the band edges. Similar results can be observed in the electronic states (Fig. 3f and Fig. S15). We also found that the electronic states were localized on the Eu(II) units isolated by organic cations in low-dimensional Eu-based halides (Fig. 3f), indicating reinforced quantum confinement effect. The emission, therefore, was attributed to 5*d*-4*f* electronic transitions of Eu(II). These results, heavy atoms (Eu), low defect states, and smaller band gap with efficient 5*d*-4*f* bandgap luminescence, contribute to vibrant radioluminescence. Thus, radioluminescence of Eu-based scintillators contains the following three stages in a modified process (Fig. 3g): (1) conversion stage, heavy atoms (Eu) under X-ray radiation generate a large of electron-hole pairs; (2) transport stage, the charge carriers mainly migrate to the conduction band (CB) Eu^2+^-5*d* and valence band (VB) Eu^2+^-4*f* without being captured at detect states (traps). (3) luminescence stage, excitonic radiative recombination at band-to-band (5*d*-4*f*) generates efficient radioluminescence.

### X-ray imaging with solution processability

**Fig. 4:**
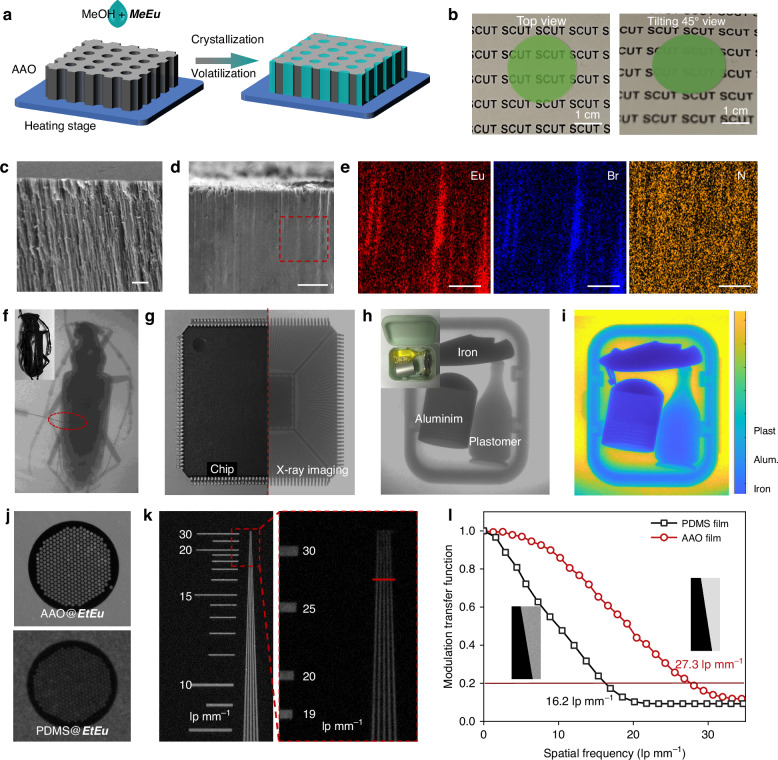
**X-ray imaging of AAO@*MeEu*** **scintillation film. a** Schematic diagram of AAO@***MeEu*** scintillation film by the suction crystallization method. **b** Photographs of AAO@***MeEu*** scintillation film at top view and tilting 45° view. **c** The cross-sectional SEM image of AAO film. **d** The cross-sectional SEM image of AAO@***MeEu*** scintillation film. **e** EDS images of Eu, Br, and N elements from the element Mapping scanning in the red dashed box of **d**. Bright-field and X-ray imaging of **f** longicorn specimen, **g** chip drive, and **h** jaffeite bottle iron knife aluminum pot hiding into plastic box. **i** Multi-color imaging through MATLAB simulation. **j** X-ray images of a copper mesh (diameter: 100 μm) using AAO@***MeEu*** and PDMS@***MeEu*** scintillation films. **k** X-ray imaging of partial region of the standard X-ray test pattern based on AAO@***MeEu***. **l** Modulation transfer function curves of the AAO@***MeEu*** and PDMS@***MeEu*** scintillation films measured by the slanted-edge method

Inspired by the promising scintillation properties of 0D ***MeEu*** halide, we further investigated the utility for X-ray imaging applications. To obtain large-area scintillation film, we take advantage of characteristics of solution processed for ***MeEu*** scintillator to incorporate into two-pass AAO matrix templates (Fig. 4a). The AAO@***MeEu*** scintillation film possess higher transparent at top view than that at tilting 45° view (Fig. 4b). The formation of the optical waveguide effect reduces optical scattering at lateral surface to enhance X-ray imaging resolution. And the waveguide structure could be further corroborated by the SEM image of AAO@***MeEu*** cross-section (Fig. 4d) compared to ones of AAO matrix templates (Fig. 4c). Besides, the EDS images with uniformly distributed Eu, Br, and N elements (Fig. 4e) form red box in Fig. 4d reveal that the ***MeEu*** is homogeneously embedded into AAO matrix templates.

A home-built X-ray imaging system integrating with X-ray source, imaging object, reflector, and COMS camera, is employed to perform X-ray imaging (Fig. S16). We demonstrate X-ray imaging with inserting a metal needle (diameter: 100 μm) into the body and clear joint structures of longicorn specimen under rate dose of 0.78 μGy s^−1^ (Fig. 4f), 60 times lower than X-ray diagnostic dosage of 5.5 μGy s^−1^ ^45^. The imaging easily distinguished interior circuit of a chip drive, as shown in Fig. 4g, emphasizing capacity of AAO@***MeEu*** film in industrial inspection. Moreover, we prepared three objects, i.e., jaffeite bottle iron knife aluminum pot, to hide into plastic box (Fig. 4h), to evaluate potential of AAO@***MeEu*** film for application in security. Clear imaging accompanied by different grayscale values can be observed for objects of different materials. Furthermore, the types of materials can be further defined by color depth through MATLAB simulation (Fig. 4i). Thanks to the low X-ray afterglow performance, the AAO@***MeEu*** film exhibit no imaging ghost in dynamic X-ray imaging compared to CsI:Tl scintillators, as shown in Fig. S17 and Video S1. In order to appraise superiority of AAO@***MeEu*** film in imaging resolution, the mixed film (PDMS@***MeEu***) by incorporating the ***MeEu*** into a polydimethylsiloxane (PDMS) was prepared as a comparison. As expected, AAO@***MeEu*** film manifest more distinct imaging of a tiny copper hexagonal mesh (diameter: 100 μm, Fig S18) than that using PDMS@ ***MeEu*** (Fig. 4j). The X-ray imaging spatial resolutions of two films are defined by a standard line-pair card (TYPE 39 b) (Fig. S19). X-ray imaging employed AAO@***MeEu*** demonstrates spatial resolution up to ~ 28 lp mm^−1^ (Fig. 4k), outclassing ~ 16.5 lp mm^−1^ of PDMS@ ***MeEu*** (Fig. S20). Additionally, we measured X-ray imaging using coupling ***MeEu*** in AAO thin films with high transparency glass substrates (thickness ~ 1 mm) and a silica template with capillary microholes (thickness ~ 1 mm)^57^. As shown in Fig. S21, X-ray imaging employed AAO@***MeEu*** with glass substrates demonstrates spatial resolution up to ~ 27 lp mm^−1^, which is almost consistent with a single AAO@***MeEu*** film, superior to ~20 lp mm^−1^ of ***MeEu*** with a silica template. As shown in Fig. 4l, the spatial resolution results can be further quantified by calculating the modulation transfer function (MTF) using slanted-edge method^58,59^. AAO@***MeEu*** owns higher spatial resolution of ~ 27.3 lp mm^−1^ at MTF = 0.2, which is superior to the commercial CsI:Tl ~ 10 lp mm^−1^ and competitive to those of reported most metal halide scintillators (Table S7).

## Discussion

In conclusion, we combined the B-site screening and molecular dimensional engineering for developing rare-earth-based halide scintillators for sensitive X-ray detection. As expected, low dimensional Eu(II)-based halides demonstrated edge-sharing for 1D and face-sharing for 0D structures, respectively. The efficient spin-allowed 5*d-*4*f* bandgap emission, lowing bandgaps and enhancing quantum confinement effect lead to the highly enhanced scintillation performance, i.e., light yield ~ 73100 ± 800 Ph MeV^−1^, detection limit 18.6 nGy s^−1^, X-ray afterglow ~1% @ 9.6 μs. In addition, the AAO@***MeEu*** scintillation film with optical waveguide effect exhibits high X-ray imaging resolution to >27 lp mm^−1^, superior to those of most commercial and metal halide scintillators, potential of applications in medical diagnosis, industrial inspection and security. Our findings offer insights for developing low-dimensional rare-earth-based halides as next-generation high-performance scintillation materials.

## Methods

### Synthesis of Eu(II)-based hybrid single crystals

***EtEu***: 1 mmol EuBr_2_ (99.5%, Grirem Advanced Materials Co. Ld.) and 1 mmol Tetraethylammonium bromide (99.7%, Alfa Aesar) were added to a 10 ml Pyrex-bottle with 3 mL anhydrous MeOH (99.7%, Alfa Aesar). The solution was stirred for 1 h at 60 °C to obtain a well-soluble solution. The viridescent single crystals were grown by slow evaporation at room temperature. After 1 week, the single crystals could be recovered and stored in the glovebox for further characterization. ***MeEu***: the similar method was used to obtain MeEu single crystals. The only difference is that 0.6 mmol EuBr_2_ and 0.5 mmol Tetramethylammonium bromide (99.7%, Alfa Aesar) were dissolved into 8 mL anhydrous MeOH. All manipulations were performed in a glovebox filled with argon where H_2_O and O_2_ levels < 0.1 ppm.

### Fabrication of *MeEu*@AAO scintillation screens

The ***MeEu*** hybrids were dissolved into MeOH. The solution was uniformly dropped onto the two-pass AAO matrix templates with pore size (interval of 60 nanometers, diameter of 30 nanometers). The solvent MeOH slowly evaporated and ***MeEu*** scintillators were embedded into pores of AAO matrix templates at room temperature. Repeat the above steps until ***MeEu*** scintillators fully filled into AAO film. All manipulations were performed in glovebox filled with argon where H_2_O and O_2_ levels <0.1 ppm.

### Supplementary information

Supplementary information for the publication

Supplementary Video for the publication

## Data Availability

The data that support the findings of this study are available from the corresponding authors upon reasonable request.
