# Supplementary material for: Hybrid Eu(II)-bromide scintillators with efficient 5d-4f bandgap transition for X-ray imaging
Source: Light Sci Appl. 2024 Aug 29;13:222. doi: 10.1038/s41377-024-01589-w (PMC11362449; doi:10.1038/s41377-024-01589-w)
Supplement: Supplementary file 1 — Supplementary information for the publication [file 41377_2024_1589_MOESM1_ESM.docx]

Supplementary Information for

**Hybrid Eu(II)-bromide scintillators with efficient 5*d*-4*f* bandgap transition for X-ray imaging**

*Kai Han^1^,* *Jiance Jin^1^, Yuzhen Wang^2^, Xinquan Zhou^1^, Yongsheng Sun^2^,*

*Lihan Chen^1^, Zhiguo Xia^1,2*^*

^1^The State Key Laboratory of Luminescent Materials and Devices, Guangdong Provincial Key Laboratory of Fiber Laser Materials and Applied Techniques, Guangdong Engineering Technology Research and Development Centre of Special Optical Fiber Materials and Devices, School of Physics and Optoelectronics, South China University of Technology, Guangzhou, 510641, China.

^2^ School of Materials Science and Engineering, South China University of Technology, Guangzhou, 510641, China.

* Corresponding authors.

[xiazg@scut.edu.cn](mailto:xiazg@scut.edu.cn) (Z. Xia)

Contents:

[Table S1 6](#_Toc174475512)

[Table S2 7](#_Toc174475513)

[Table S3 9](#_Toc174475514)

[Table S4 13](#_Toc174475515)

[Table S5 14](#_Toc174475516)

[Table S6 15](#_Toc174475517)

[Table S7 16](#_Toc174475518)

[Fig. S1 17](#_Toc174475519)

[Fig. S2 18](#_Toc174475520)

[Fig. S3 19](#_Toc174475521)

[Fig. S4 20](#_Toc174475522)

[Fig. S5 21](#_Toc174475523)

[Fig. S6 22](#_Toc174475524)

[Fig. S7 23](#_Toc174475525)

[Fig. S8 24](#_Toc174475526)

[Fig. S9 25](#_Toc174475527)

[Fig. S10 26](#_Toc174475528)

[Fig. S11 27](#_Toc174475529)

[Fig. S12 28](#_Toc174475530)

[Fig. S13 29](#_Toc174475531)

[Fig. S14 30](#_Toc174475532)

[Fig. S15 31](#_Toc174475533)

[Fig. S16 32](#_Toc174475534)

[Fig. S17 33](#_Toc174475535)

[Fig. S18 34](#_Toc174475536)

[Fig. S19 35](#_Toc174475537)

[Fig. S20 36](#_Toc174475538)

[Fig. S21 37](#_Toc174475539)

[References 38](#_Toc174475540)

**Measurements**

**Structural Characterization** **and microscopy.** The crystal structure determination was carried out by a XtaLAB Synergy R X-ray single-crystal diffractometer with a hybrid pixel array detector and a Mo Kα radiation source (λ = 0.71073 Å) at 150 K. The structures were solved by direct methods and refined by full-matrix least-squares on *F*^2^ using the *SHELX*-2018 program package. The crystallographic data and details of structural refinements are listed in Table S1 CCDC number 2314513 for ***MeEu*** and 2314514 for ***EtEu*** contain the supplementary crystallographic data for this paper. These data can be obtained free of charge from The Cambridge Crystallographic Data Centre via [www.ccdc.cam.ac.uk/data_request/cif](http://www.ccdc.cam.ac.uk/data_request/cif). The phase purity were determined at room temperature using a D8 Advance diffractometer with Cu Kα (λ = 1.541862 Å) radiation operating at 40 kV and 15 mA. The elemental composition was determined using X-ray photoelectron spectroscopy (XPS, ESCALAB 250Xi, Thermo Scientific Inc., USA) with a monochromatic Al Kα source. Field-emission scanning electron microscope (FE-SEM, Quanta F250, FEI, USA) was used to characterize the elemental composition and morphologies. The EPR spectra of the samples were measured using Bruker EMX-plus X-band EPR, and the experimental parameters were a microwave frequency of 9.8 GHz, a microwave power of 0.8 mW, a magnetic field modulation amplitude of 0.5 G, a magnetic field modulation frequency of 100 kHz, and a time constant of 30 ms.

**Photoluminescence studies.** Absorption spectra were recorded using a using a SHIMADZU 2600 UV–vis spectrophotometer. Steady-state photoluminescence excitation (PLE) and emission (PL) of the samples were measured through FLS1000 fluorescence spectrophotometer (Edinburgh Instruments). PLE and PL spectra were obtained using a 450 W xenon lamp as the excitation source; while decay curves were gained via 340 nm pulse laser diode as the excitation source. Photoluminescence quantum yield test was performed at room temperature by using FLS1000 fluorescence spectrophotometer with an integrated sphere (F-M01). The integrating sphere consists of a 120 mm inner diameter spherical cavity, which is machined from a PTFE like material with a reflectance >99% over the spectral range of 400 nm-1600 nm. The PLQY was calculated by using the following equations:

$\eta_{PLQY}=\frac{\varepsilon}{\alpha}=\frac{\int L_{S}}{\int E_{R}-\int E_{S}}$ (1)

where *ε* is the number of photons emitted by the sample, and *α* is the number of photons absorbed by the sample. *L_S_* is the luminescence emission spectrum of the sample; *E_S_* is the spectrum of the light used for exciting the sample; *E_R_* is the spectrum of the excitation light without the sample in the sphere. The monochromatic light filtered from a xenon lamp is selected as the excitation light. In detail, the optimal excitation wavelength (400 nm, 400 nm and 450 nm from monochromatic light filtered from a xenon lamp) are used as the excitation light for CsEuBr_3_, ***EtEu*** and ***MeEu*** hybrids. All the measured PLQY values in this work were measured via a statistical average value for at least 3 times, as shown in supporting information (Table S5). Thermoluminescence curve measurements were performed with a TL dosimeter (FJ-427A1) from 300 to 500 K. The sample was pre-irradiated by X-ray for 10 min at room temperature.,

**Radioluminescence studies.** RL measurements were carried out at room temperature using a homemade system with an X-ray tube (Mini-X, Amptek; Mo target; tube voltage, 50 kV) an integrating sphere and a spectrometer (FLS1000, Edinburgh). A commercial LuAG:Ce scintillator (light yield, ~25000 Ph MeV^–1^) was used as the reference to caculate LY of ***EtEu*** and ***MeEu*** scintillators. In our case, we calculated the relative LY by integrating the X-ray-induced RL spectra multiply by c X-ray attenuation efficiencies at same thickness of 500 μm.

The caculated result was cross-checked with CsEuBr_3_ obtaining a light yield of 21000 Ph MeV^–1^, which matches reported value (~21000 Ph MeV^–1^) and proves the validity of the measurement method. By measuring the dose-dependent RL spectra, a linear relationship between the RL intensity against dose rate was obtained, where the slope k represented the sensitivity of the X-ray detector. Then, the noise intensity was recorded under the same measurement conditions with none samples. The noise intensity was analysed and fitted by the Gaussian function, where the full-width at half-maximum (FWHM) was used as the average noise. The detection limit (DL) was obtained as DL = 3 × FWHM/k. The X-ray imaging system was constructed as shown in Supplementary Figure 16. A CMOS camera (KnightCam S455) with 9568 × 6380 pixels and a 3.76-μm pixel size and the lens (Canon, EF 100 mm f/2.8L IS USM) were used to collect the X-ray images, and the exposure time was set to 30 s. A leakage-and-low-level X-ray ion chamber dose meter (10X5-180, Radcal) was used to calibrate the dose rates under changing the tube current and the distance between tube and object.

**Calculation method.** DFT calculations of CsEuBr_3_, ***EtEu*** and ***MeEu*** were performed using projector augmented wave (PAW) method based on a generalized gradient approximation (GGA), and the Perdew–Burke–Ernzerhof (PBE) format was adopted for the exchange correlation potential for structural relaxation.^1,2^ The cutoff energy of a plane-wave basis was set as 520 eV. Monkhorst–Pack mesh of k-point was set as 3 × 3 × 3 for CsEuBr_3_, 3 × 3 × 2 for ***EtEu*** and 3 × 1 × 2 for ***MeEu***, respectively. The atoms in each compound were fully relaxed until the Hellmann–Feynman forces on them were within 0.01 eV Å^−1^. The electronic iteration convergence was set as 10^−5^ eV. To consider the strong Coulomb repulsion for the Eu-4*f* electrons, we use a Hubbard correction to PBE (GGA+U).^3^

Table S1 Crystallographic data and refinement details for ***EtEu*** and ***MeEu*** hybrids.

| Parameter | ***MeEu*** | ***EtEu*** |
| --- | --- | --- |
| Empirical formula | C26H80Br16Eu5N6O2 | C9H24Br3EuNO |
| Formula Mass | 2547.32 | 553.98 |
| Crystal system | Monoclinic | Monoclinic |
| Space group | *P2_1/n_* | *P2_1/n_* |
| *a*/Å | 9.2516(3) | 10.1146(3) |
| *b*/Å | 21.7058(5) | 8.9212(3) |
| *c*/Å | 16.7838(4) | 18.6171(6) |
| *α/°* | 90 | 90 |
| *β/°* | 91.504(2) | 92.696(2) |
| *γ/°* | 90 | 90 |
| *V*/Å^3^ | 3369.25(16) | 1678.04(9) |
| *Z* | 2 | 4 |
| *T*/K | 150(2) | 150(2) |
| *λ*/Å | 0.71073 | 0.71073 |
| *F*(000) | 2338 | 1044 |
| *ρ*_calcd_/g cm^-3^ | 2.511 | 2.193 |
| *μ*/mm^-1^ | 14.100 | 10.863 |
| Measured refls. | 32579 | 15783 |
| Independent refls. | 5934 | 2963 |
| No. of parameters | 264 | 142 |
| *R*_int_ | 0.0677 | 0.0908 |
| *R*_1_ (*I*> 2*σ*(*I*))^a^ | 0.0362 | 0.0376 |
| *wR*(*F*^2^) (*I*> 2*σ*(*I*))^b^ | 0.0787 | 0.0931 |
| GOF | 1.051 | 1.091 |

Table S2 Selected bond lengths and angles for ***EtEu*** hybrids.

| Bond /Angle | Value (Å) / (°) |
| --- | --- |
| Eu(1)-O(1) | 2.558(4) |
| Eu(1)-Br(3) | 2.9964(6) |
| Eu(1)-Br(2) | 3.0271(5) |
| Eu(1)-Br(1) | 3.0344(5) |
| Eu(1)-Br(1)#1 | 3.0383(5) |
| Eu(1)-Br(2)#2 | 3.0561(5) |
| Eu(1)-Eu(1)#1 | 4.48534(16) |
| Eu(1)-Eu(1)#2 | 4.48539(16) |
|  |  |
| O(1)-Eu(1)-Br(3) | 175.87(8) |
| O(1)-Eu(1)-Br(2) | 83.74(8) |
| Br(3)-Eu(1)-Br(2) | 98.192(17) |
| O(1)-Eu(1)-Br(1) | 88.92(8) |
| Br(3)-Eu(1)-Br(1) | 94.489(16) |
| Br(2)-Eu(1)-Br(1) | 95.942(15) |
| O(1)-Eu(1)-Br(1)#1 | 81.91(8) |
| Br(3)-Eu(1)-Br(1)#1 | 94.683(16) |
| Br(2)-Eu(1)-Br(1)#1 | 82.677(14) |
| Br(1)-Eu(1)-Br(1)#1 | 170.828(11) |
| O(1)-Eu(1)-Br(2)#2 | 87.52(8) |
| Br(3)-Eu(1)-Br(2)#2 | 90.625(16) |
| Br(2)-Eu(1)-Br(2)#2 | 171.123(11) |
| Br(1)-Eu(1)-Br(2)#2 | 82.261(14) |
| Br(1)#1-Eu(1)-Br(2)#2 | 97.706(15) |
| O(1)-Eu(1)-Eu(1)#1 | 69.29(7) |
| Br(3)-Eu(1)-Eu(1)#1 | 109.697(13) |
| Br(2)-Eu(1)-Eu(1)#1 | 42.747(10) |
| Br(1)-Eu(1)-Eu(1)#1 | 133.201(13) |
| Br(1)#1-Eu(1)-Eu(1)#1 | 42.354(10) |
| Br(2)#2-Eu(1)-Eu(1)#1 | 134.518(14) |
| O(1)-Eu(1)-Eu(1)#2 | 98.69(7) |
| Br(3)-Eu(1)-Eu(1)#2 | 82.349(11) |
| Br(2)-Eu(1)-Eu(1)#2 | 137.837(13) |
| Br(1)-Eu(1)-Eu(1)#2 | 42.422(10) |
| Br(1)#1-Eu(1)-Eu(1)#2 | 139.470(13) |
| Br(2)#2-Eu(1)-Eu(1)#2 | 42.245(10) |
| Eu(1)#1-Eu(1)-Eu(1)#2 | 167.954(14) |
| Eu(1)-Br(1)-Eu(1)#2 | 95.224(14) |
| Eu(1)-Br(2)-Eu(1)#1 | 95.007(15) |
| C(9)-O(1)-Eu(1) | 122.1(3) |
| C(9)-O(1)-H(1) | 109.5 |
| Eu(1)-O(1)-H(1) | 110.7 |
| Symmetry transformations used to generate equivalent atoms:  #1 -*x*+1/2,*y*+1/2,-*z*+1/2 #2 -*x*+1/2,*y*-1/2,-*z*+1/2 | |

Table S3 Selected bond lengths and angles for ***MeEu*** hybrids.

| Bond /Angle | Value (Å) / (°) |
| --- | --- |
| Eu(1)-Br(1) | 3.0143(6) |
| Eu(1)-Br(1)#1 | 3.0143(6) |
| Eu(1)-Br(2)#1 | 3.0245(7) |
| Eu(1)-Br(2) | 3.0245(7) |
| Eu(1)-Br(3) | 3.0517(7) |
| Eu(1)-Br(3)#1 | 3.0518(7) |
| Eu(1)-Eu(2)#1 | 3.9379(3) |
| Eu(1)-Eu(2) | 3.9380(3) |
| Eu(2)-Br(5) | 3.0136(8) |
| Eu(2)-Br(4) | 3.0172(7) |
| Eu(2)-Br(6) | 3.0187(7) |
| Eu(2)-Br(1) | 3.0217(7) |
| Eu(2)-Br(2) | 3.0507(8) |
| Eu(2)-Br(3) | 3.0552(8) |
| Eu(2)-Eu(3) | 3.9767(4) |
| Eu(3)-O(1) | 2.488(5) |
| Eu(3)-Br(7) | 2.9671(8) |
| Eu(3)-Br(8) | 2.9893(7) |
| Eu(3)-Br(5) | 3.0438(8) |
| Eu(3)-Br(6) | 3.1202(7) |
| Eu(3)-Br(4) | 3.1772(7) |
|  |  |
| Br(1)-Eu(1)-Br(1)#1 | 180.0 |
| Br(1)-Eu(1)-Br(2)#1 | 99.120(19) |
| Br(1)#1-Eu(1)-Br(2)#1 | 80.880(19) |
| Br(1)-Eu(1)-Br(2) | 80.880(19) |
| Br(1)#1-Eu(1)-Br(2) | 99.120(19) |
| Br(2)#1-Eu(1)-Br(2) | 180.0 |
| Br(1)-Eu(1)-Br(3) | 84.441(19) |
| Br(1)#1-Eu(1)-Br(3) | 95.559(19) |
| Br(2)#1-Eu(1)-Br(3) | 97.33(2) |
| Br(2)-Eu(1)-Br(3) | 82.67(2) |
| Br(1)-Eu(1)-Br(3)#1 | 95.558(19) |
| Br(1)#1-Eu(1)-Br(3)#1 | 84.442(19) |
| Br(2)#1-Eu(1)-Br(3)#1 | 82.67(2) |
| Br(2)-Eu(1)-Br(3)#1 | 97.33(2) |
| Br(3)-Eu(1)-Br(3)#1 | 180.0 |
| Br(1)-Eu(1)-Eu(2)#1 | 130.643(13) |
| Br(1)#1-Eu(1)-Eu(2)#1 | 49.357(13) |
| Br(2)#1-Eu(1)-Eu(2)#1 | 49.884(14) |
| Br(2)-Eu(1)-Eu(2)#1 | 130.116(14) |
| Br(3)-Eu(1)-Eu(2)#1 | 130.116(14) |
| Br(3)#1-Eu(1)-Eu(2)#1 | 49.884(14) |
| Br(1)-Eu(1)-Eu(2) | 49.358(13) |
| Br(1)#1-Eu(1)-Eu(2) | 130.642(13) |
| Br(2)#1-Eu(1)-Eu(2) | 130.116(14) |
| Br(2)-Eu(1)-Eu(2) | 49.883(14) |
| Br(3)-Eu(1)-Eu(2) | 49.884(14) |
| Br(3)#1-Eu(1)-Eu(2) | 130.116(14) |
| Eu(2)#1-Eu(1)-Eu(2) | 180.0 |
| Br(5)-Eu(2)-Br(4) | 82.45(2) |
| Br(5)-Eu(2)-Br(6) | 88.64(2) |
| Br(4)-Eu(2)-Br(6) | 80.30(2) |
| Br(5)-Eu(2)-Br(1) | 95.76(2) |
| Br(4)-Eu(2)-Br(1) | 95.28(2) |
| Br(6)-Eu(2)-Br(1) | 173.33(2) |
| Br(5)-Eu(2)-Br(2) | 170.13(2) |
| Br(4)-Eu(2)-Br(2) | 106.86(2) |
| Br(6)-Eu(2)-Br(2) | 96.09(2) |
| Br(1)-Eu(2)-Br(2) | 80.34(2) |
| Br(5)-Eu(2)-Br(3) | 88.42(2) |
| Br(4)-Eu(2)-Br(3) | 170.77(2) |
| Br(6)-Eu(2)-Br(3) | 100.91(2) |
| Br(1)-Eu(2)-Br(3) | 84.26(2) |
| Br(2)-Eu(2)-Br(3) | 82.18(2) |
| Br(5)-Eu(2)-Eu(1) | 121.583(16) |
| Br(4)-Eu(2)-Eu(1) | 135.261(16) |
| Br(6)-Eu(2)-Eu(1) | 131.795(16) |
| Br(1)-Eu(2)-Eu(1) | 49.193(13) |
| Br(2)-Eu(2)-Eu(1) | 49.304(13) |
| Br(3)-Eu(2)-Eu(1) | 49.808(13) |
| Br(5)-Eu(2)-Eu(3) | 49.294(15) |
| Br(4)-Eu(2)-Eu(3) | 51.851(14) |
| Br(6)-Eu(2)-Eu(3) | 50.749(14) |
| Br(1)-Eu(2)-Eu(3) | 129.729(16) |
| Br(2)-Eu(2)-Eu(3) | 139.434(17) |
| Br(3)-Eu(2)-Eu(3) | 122.073(17) |
| Eu(1)-Eu(2)-Eu(3) | 170.223(11) |
| O(1)-Eu(3)-Br(7) | 92.13(12) |
| O(1)-Eu(3)-Br(8) | 94.62(14) |
| Br(7)-Eu(3)-Br(8) | 99.08(2) |
| O(1)-Eu(3)-Br(5) | 168.37(13) |
| Br(7)-Eu(3)-Br(5) | 93.85(2) |
| Br(8)-Eu(3)-Br(5) | 94.27(2) |
| O(1)-Eu(3)-Br(6) | 83.38(14) |
| Br(7)-Eu(3)-Br(6) | 93.97(2) |
| Br(8)-Eu(3)-Br(6) | 166.87(2) |
| Br(5)-Eu(3)-Br(6) | 86.27(2) |
| O(1)-Eu(3)-Br(4) | 93.01(12) |
| Br(7)-Eu(3)-Br(4) | 168.42(2) |
| Br(8)-Eu(3)-Br(4) | 90.84(2) |
| Br(5)-Eu(3)-Br(4) | 79.38(2) |
| Br(6)-Eu(3)-Br(4) | 76.337(19) |
| O(1)-Eu(3)-Eu(2) | 119.81(13) |
| Symmetry transformations used to generate equivalent atoms:  #1 -*x*+1,-*y*+1,-*z*+1 | |

Table S4 Photoluminescence parameters of 3D CsEuBr_3_, 1D ***EtEu*** and 0D ***MeEu*** hybrids.

| Composition | Excitation max.  (nm) | Emission max.  (nm) | FWHM  (nm) |
| --- | --- | --- | --- |
| CsEuBr_3_ | 430 | 445 | 27 |
| ***EtEu*** | 420 | 450 | 26 |
| ***MeEu*** | 450 | 500 | 40 |

Table S5 The calculated and experimented photoluminescence parameters of CsEuBr_3_, ***EtEu*** and ***MeEu*** hybrids.

| Composition | Experiment | | | | | Calculation | | | | | |
| --- | --- | --- | --- | --- | --- | --- | --- | --- | --- | --- | --- |
|  | Abs (eV) | | Em (eV) | | Δ*S* (eV) | Abs (eV) | | Em (eV) | | Δ*S* (cm^-1^) | |
| CsEuBr_3_ | 2.88 | 2.79 | | 0.09 | | | 3.002 | | 2.897 | | 0.105 |
| ***EtEu*** | 2.95 | 2.76 | | 0.19 | | | 2.825 | | 2.707 | | 0.114 |
| ***MeEu*** | 2.76 | 2.48 | | 0.28 | | | 2.684 | | 2.417 | | 0.261 |

**Note:**

Following Dorenbos’ semiempirical model, the energy of the ﬁrst allowed 4*f* → 5*d* transition of the free RE ion is lowered by the crystalline environment, with a shift denoted D(A). This lowering is the sum of the spectroscopic redshift arising from the centroid shift of the RE_5_*_d_* energy, *ε*_c_(A), and the crystal-ﬁeld splitting, *ε*_cfs_(A), of the RE_5_*_d_* states. the related work for the Eu^2+^ doped phosphors is limited because of the more complex electronic conﬁguration of the Eu^2+^ ion. However, a similar quantitative expression for the Eu^2+^ ion can be expected since these two ions have similar 4*f* → 5*d* neutral excitations.^4^ A semiempirical relationship between the redshift for the 5d state of Eu^2+^ and Ce^3+^ ions, in the same host, has been determined:^5^

$D\left( \mathrm{Eu}^{2+},A \right)=0.64\times D\left( \mathrm{Ce}^{3+},A \right)-0.233Ev$ (2)

This equation indicates that the redshift of the 5*d* state of the Eu^2+^ ion is smaller than the one of the Ce^3+^ ion. The difference in slope by a factor 0.64 might be due to the effect of the remaining six Eu 4*f* electrons in the excited state and the intercept of 0.233 might be due to the different ionic radius of the two ions. A direct expression for the Eu^2+^ ion in compound was proposed:^6^

$D\left( \mathrm{Eu}^{2+},A \right)=\varepsilon_{c}\left( \mathrm{Eu}^{2+},A \right)+\frac{\varepsilon_{cfs}\left( \mathrm{Eu}^{2+},A \right)}{r\left( \mathrm{Eu}^{2+},A \right)}-1890{\text{ }\mathrm{cm}}^{-1}$ (3)

Where

$\varepsilon_{c}\left( \mathrm{Eu}^{2+},A \right)=1.44\times0.64\times{10}^{17}\sum_{i=1}^{N} \frac{\alpha_{sp}^{i}}{R_{i}^{6}}$ (4)

$\varepsilon_{\mathrm{cfs}}=0.64\times\frac{\beta}{R_{av}^{2}}$ (5)

Then, the redshift of Eu 5*d* state can be determined with available ﬁrst-principles geometry information on the ground and excited states. Finally, the transition energy of Eu^2+^ ion in compound A is

$E_{abs}\left( \mathrm{Eu}^{2+},A \right)=4.19 eV-D\left( \mathrm{Eu}^{2+},A \right)$ (6)

where the ﬁrst 4*f* → 5*d* transition energy of 4.19 eV (34 004 cm^−1^).^5,7^

The 5*d* →4*f* emission of Eu^2+^ ion is expressed by the redshift D(Eu^2+^, A) and the Stokes shift Δ*S*.^8^

$E_{em}\left( \mathrm{Eu}^{2+},A \right)=4.19 eV-D\left( \mathrm{Eu}^{2+},A \right)-\Delta S$ (7)

$\Delta S=(2S-1)\hbar\omega$ (8)

where *ħω* is the energy diﬀerence between the consecutive vibrational states (phonon energy), the Huang–Rhys factor *S* gives information about the coupling of electronic and vibrational states (i.e., electron–phonon coupling).^9^

Table S6 Photoluminescent internal/external quantum efficiency (*Փ*_IQE_ and *Փ*_EQE_) of 3D CsEuBr_3_, 1D ***EtEu*** and 0D ***MeEu*** hybrids.

| Composition | | 1 | 2 | 3 | Ave. |
| --- | --- | --- | --- | --- | --- |
| CsEuBr_3_ | *Փ*_IQE_ | 72.7 | 79.4 | 75.4 | 75.5 |
|  | *Փ*_EQE_ | 37.3 | 41.6 | 41.1 | 40.0 |
| ***EtEu*** | *Փ*_IQE_ | 81.3 | 80.5 | 77.6 | 79.8 |
|  | *Փ*_EQE_ | 40.8 | 45.3 | 48.0 | 44.7 |
| ***MeEu*** | *Փ*_IQE_ | 99.2 | 99.7 | 93.9 | 97.6 |
|  | *Փ*_EQE_ | 56.7 | 56.8 | 59.6 | 57.7 |

**Note:** The *Փ*_EQE_ is equal to the internal efficiency (*Փ*_IQE_) multiplied by the absorption coefficient (Abs.).

Table S7 Summary of the properties related to X-ray detection and imaging of conventional scintillators, other reported metal halide scintillators, organic molecule scintillators, and the ***MeEu*** hybrid scintillator (this work).

| Materials | Light yield  (Ph MeV^-1^) | Detect limit (nGy s^-1^) | Imaging spatial resolution  (lp mm^-1^) | Ref. |
| --- | --- | --- | --- | --- |
| CsPbBr_3_ | - | 17 | 0.72 at 2 lp/mm | *Nature* 2018, 561, 88-93. |
| CsPbBr_3_:1.5% Eu (in GC) | 10100 | - | 15 | *Adv. Sci.* 2021, 8, 2003728. |
| CsPbBr_3_:Lu^3+^ NCs (in GC) | ~ 7500 | 50 | 16.8 | *Adv. Mater.* 2021, 33, 2102529. |
| Cs_3_Cu_2_I_5_ NCs | 79 279 | - | ~ 1.6 | *Adv. Sci.* 2020, 7, 2000195. |
| TPP_2_MnBr_4_ | 78000 | 8 | ~ 15.7 | *Adv. Mater.* 2022, 34, 2110420. |
| Cs2ZrCl6:Te | 53000 | 56 | 14.6 | *Laser Photonics Rev*. 2022, 17, 2200458. |
| Rb_2_CuBr_3_ | 91056 | 121.5 | - | *Adv. Mater.* 2019, 31, 1904711. |
| BA_10_EuI_12_ | 27000 | 5.83 | 8.95 | *Research.* 2023, 6, 0125. |
| TADF-Br | - | - | 19.8 | *Matter.* 2023, 6, 217-225. |
| Cu_2_Au_2_(R-BTT)_4_ | 17600 | 111.7 | 28.4 | *J. Am. Chem. Soc.* 2023, 145, 13816-13827. |
| DMAc-TRZ (in SO) | 73500 | 103.2 | 16.6 | *Nat. Mater.* 2022, 21, 210-216. |
| o-ITC (in PDMS) | - | 33 | - | *Nat. Photon.* 2021, 15, 187-192. |
| CsI:Tl single crystals | 64000 | - | 10 | . *IEEE Trans. Nucl. Sci.* 1997, 1, 226-230. |
| ***MeEu*@AAO** | **73100** | **18.6** | **27.3** | **This work** |


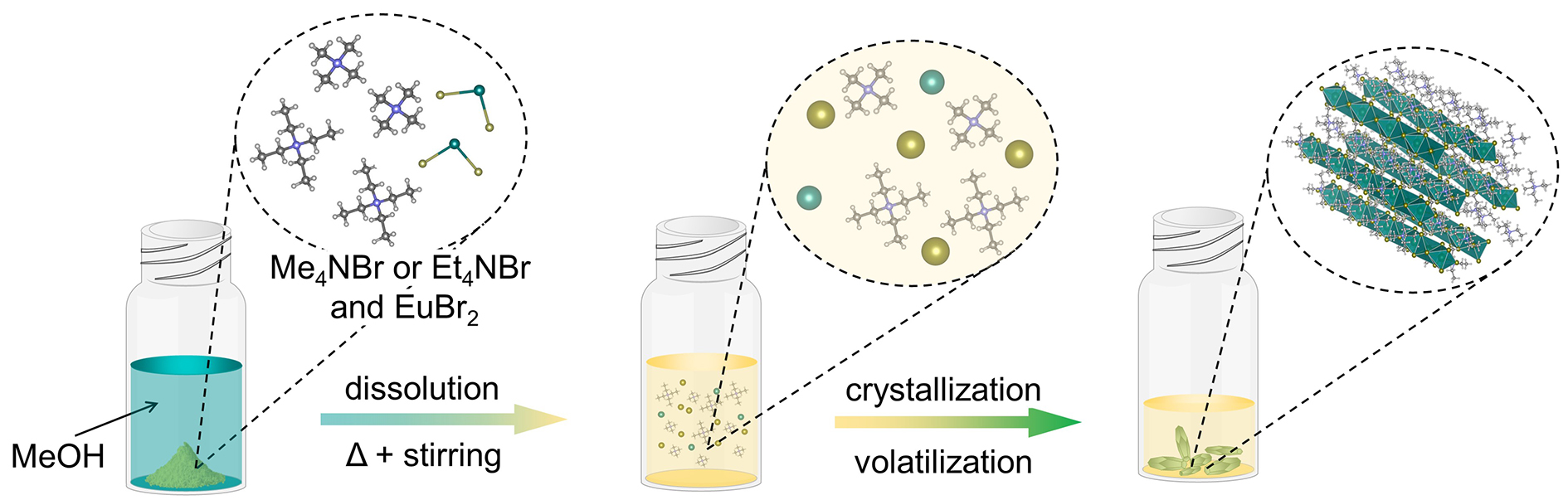


Fig. S1 **Schematic diagram of synthesis of 1D *EtEu* and 0D *MeEu* single crystals.**


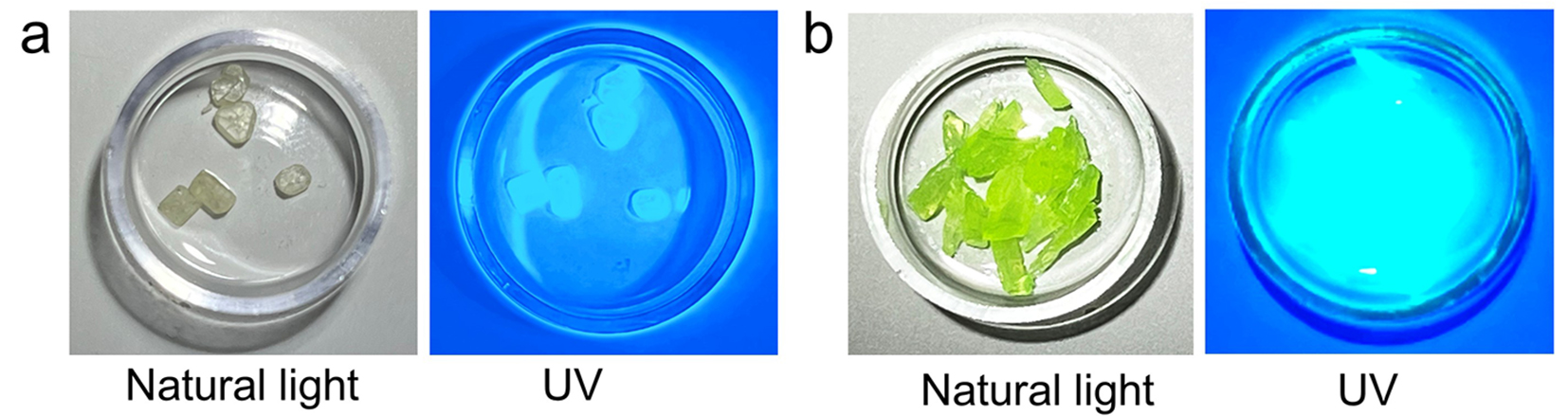


Fig. S2 **Photographs 1D *EtEu* and 0D *MeEu* single crystals. a** Photographs 1D ***EtEu*** under natural light and UV light. **b** Photographs 0D ***MeEu*** under natural light and UV light.


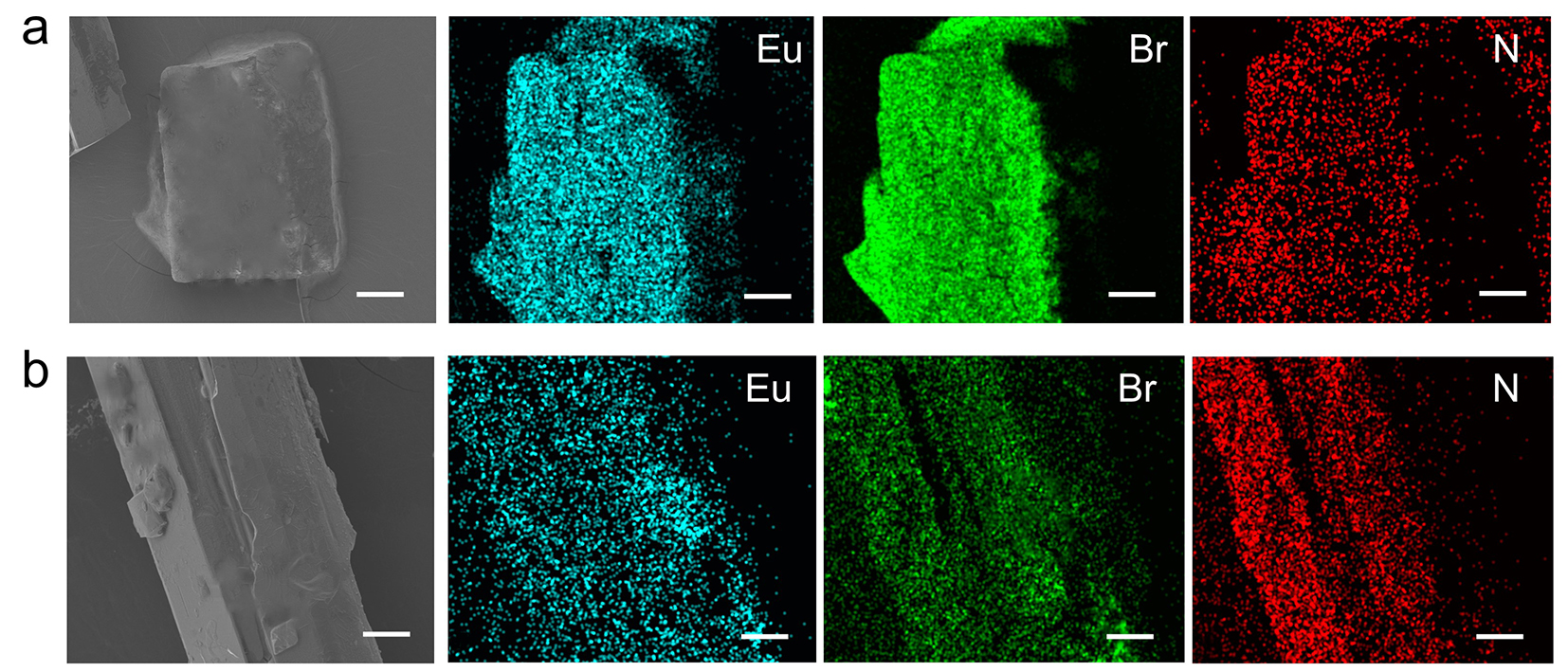


Fig. S3 **SEM-EDS of a 1D *EtEu* b 0D *MeEu* hybrids.** The ***EtEu*** hybrid exhibits uniform distribution of N, Br and Eu elements corresponding to red, green, and blue, respectively, with a scale bar of 10 μm.


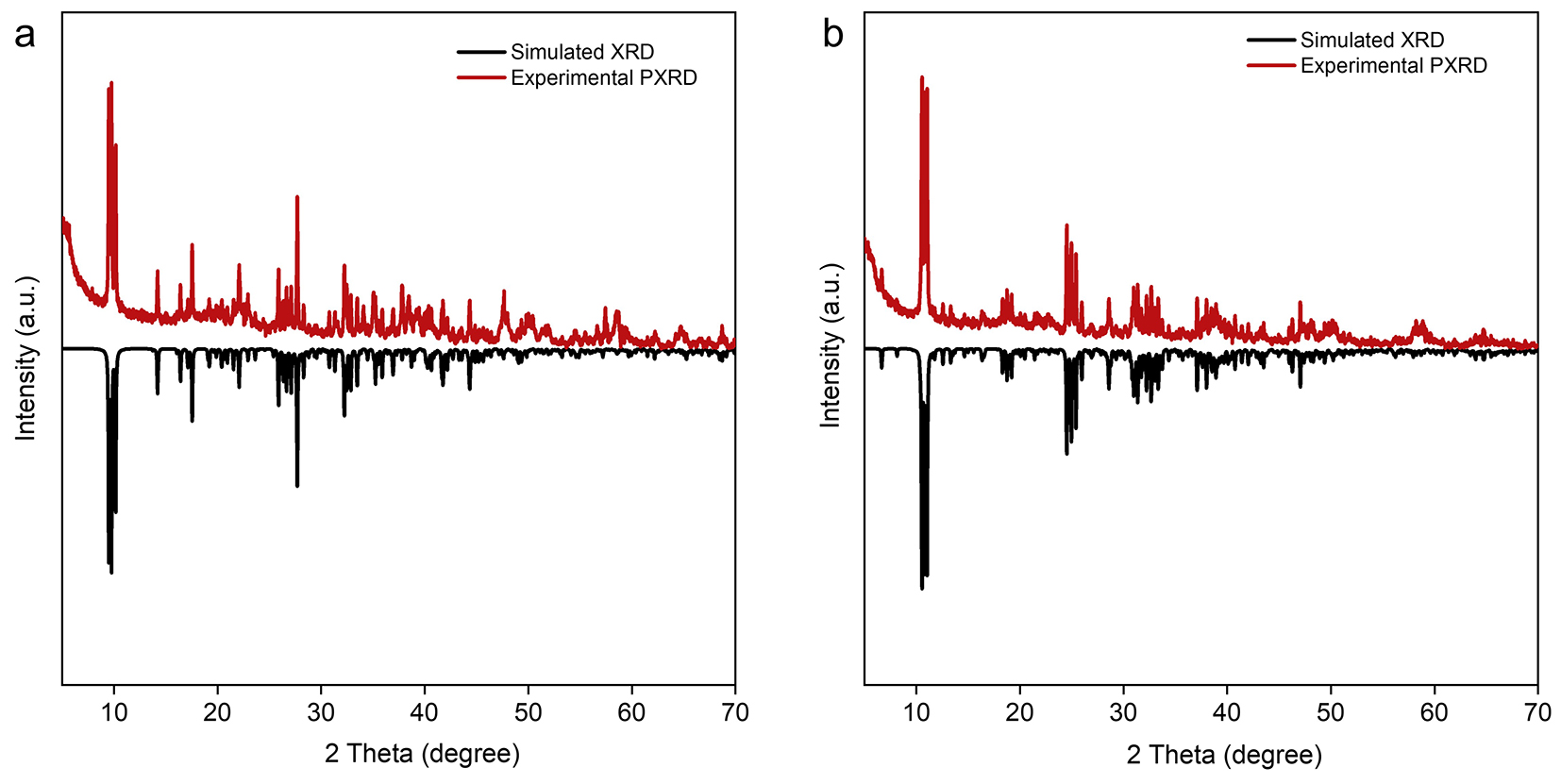


Fig. S4 **The XRD patterns.** The simulated and experimental XRD patterns of the **a** 1D ***EtEu*** **b** 0D ***MeEu*** hybrids.


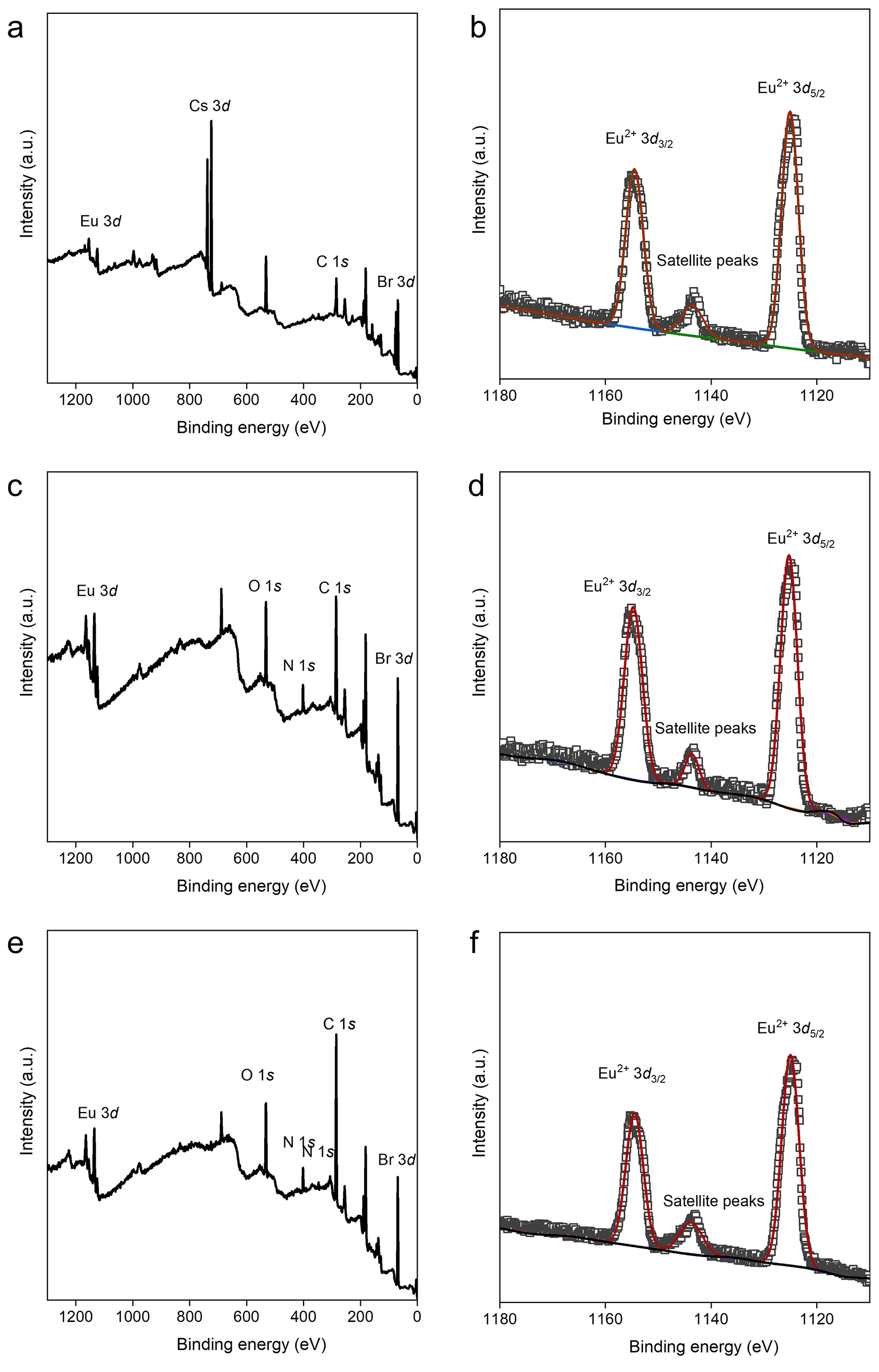


Fig. S5 **XPS spectra. a** XPS spectra of the CsEuBr_3_ and **b** High-resolution XPS spectra of Eu 3*d*. **c** XPS spectra of the 1D ***EtEu*** hybrids and **d** High-resolution XPS spectra of Eu 3*d*. **e** XPS spectra. of the 0D ***MeEu*** hybrids and **f** High-resolution XPS spectra of Eu 3*d*.

**Note:** The peaks at 1124.8 and 1154.4 eV, assigned to Eu^2+^ 3d_5/2_ and Eu^2+^ 3d_3/2_.^10^


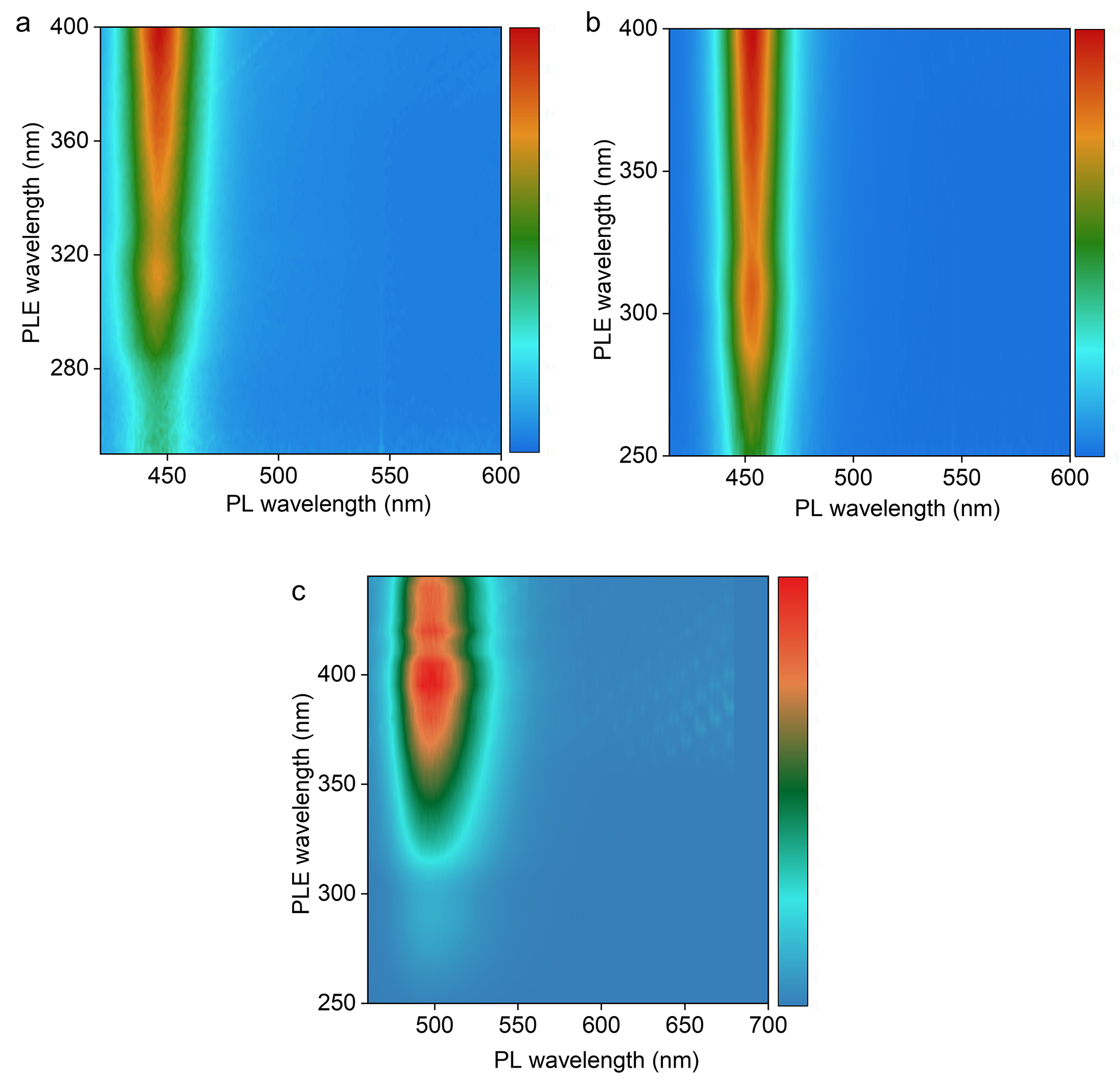


Fig. S6 **The PLE/PL correlation maps at room temperature.** Under a wider excitation band with 250-450 nm, **a** CsEuBr_3_, **b** ***EtEu*** and **b *MeEu*** hybrids only exhibit a single narrow band green emission without peak shift.


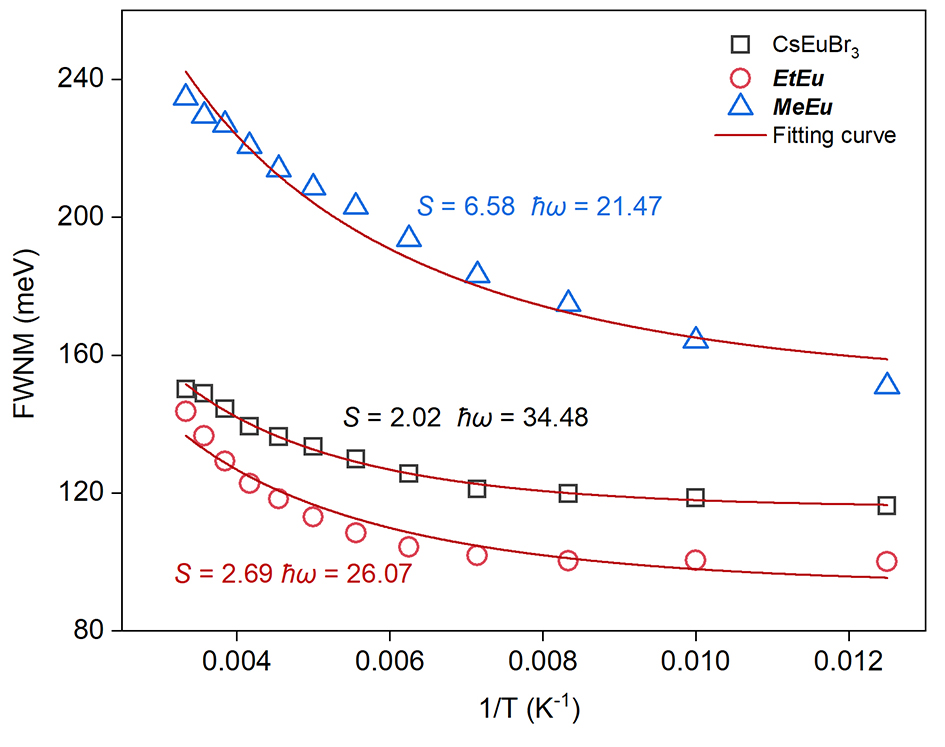


Fig. S7 Fitting results of the FWHM of the PL spectra as a function of temperatures.


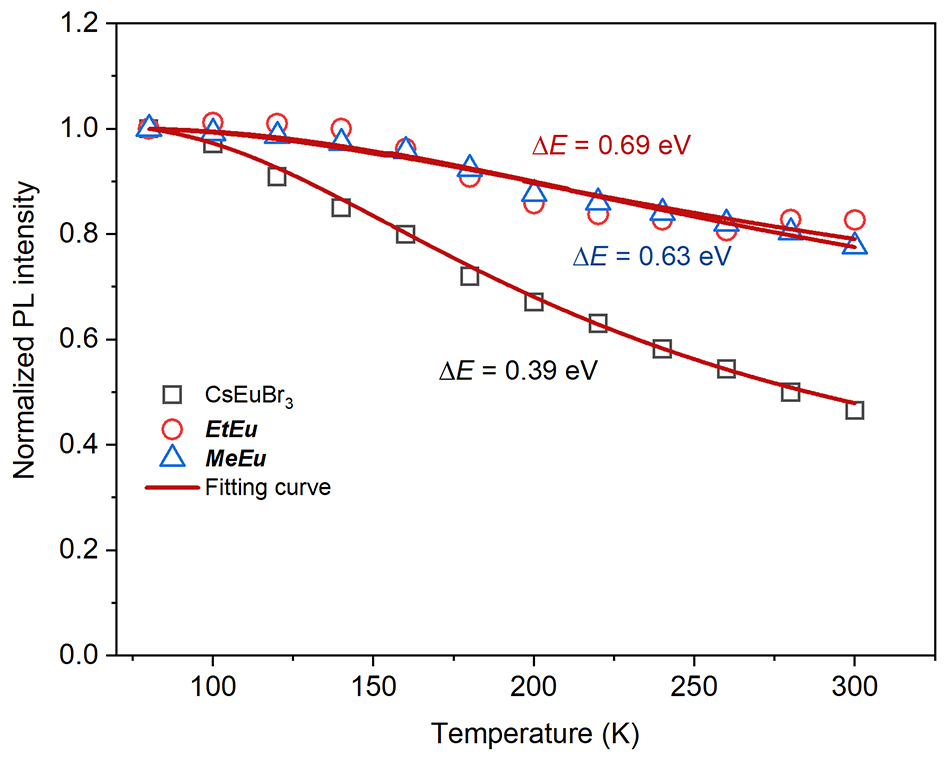


Fig. S8 Fitting results of the PL intensity as a function of temperatures.

**Note:**

Generally, the most widely used mechanism of thermal quenching is described by a simple equation:

$I_{T}=\frac{I_{0}}{1+\frac{\Gamma_{0}}{\Gamma_{\nu}}exp\left( \frac{-\Delta E}{k_{B}T} \right)}$ (9)

Where *I_T_* and *I*_0_ are the emission intensities of the phosphor at different experimental temperatures and the initial emission intensity at *T* = 0 K, respectively. *Г_v_* and *Г*_0_ represents the decay rate of radiative transition and the attempt rate for the thermal quenching process, respectively. Δ*E* denotes the energy barrier of thermal quenching and *k*_B_ is Boltzmann's constant. The thermal quenching barrier Δ*E* could be further derived through:^11^

$\Delta E=\frac{T_{0.5}}{680}$ (10)

where *T*_0.5_ is defined as the quenching temperature at which the emission intensity decreases to 50% of its initial intensity. Obviously, a high Δ*E* value means a good thermal stability of the phosphor.


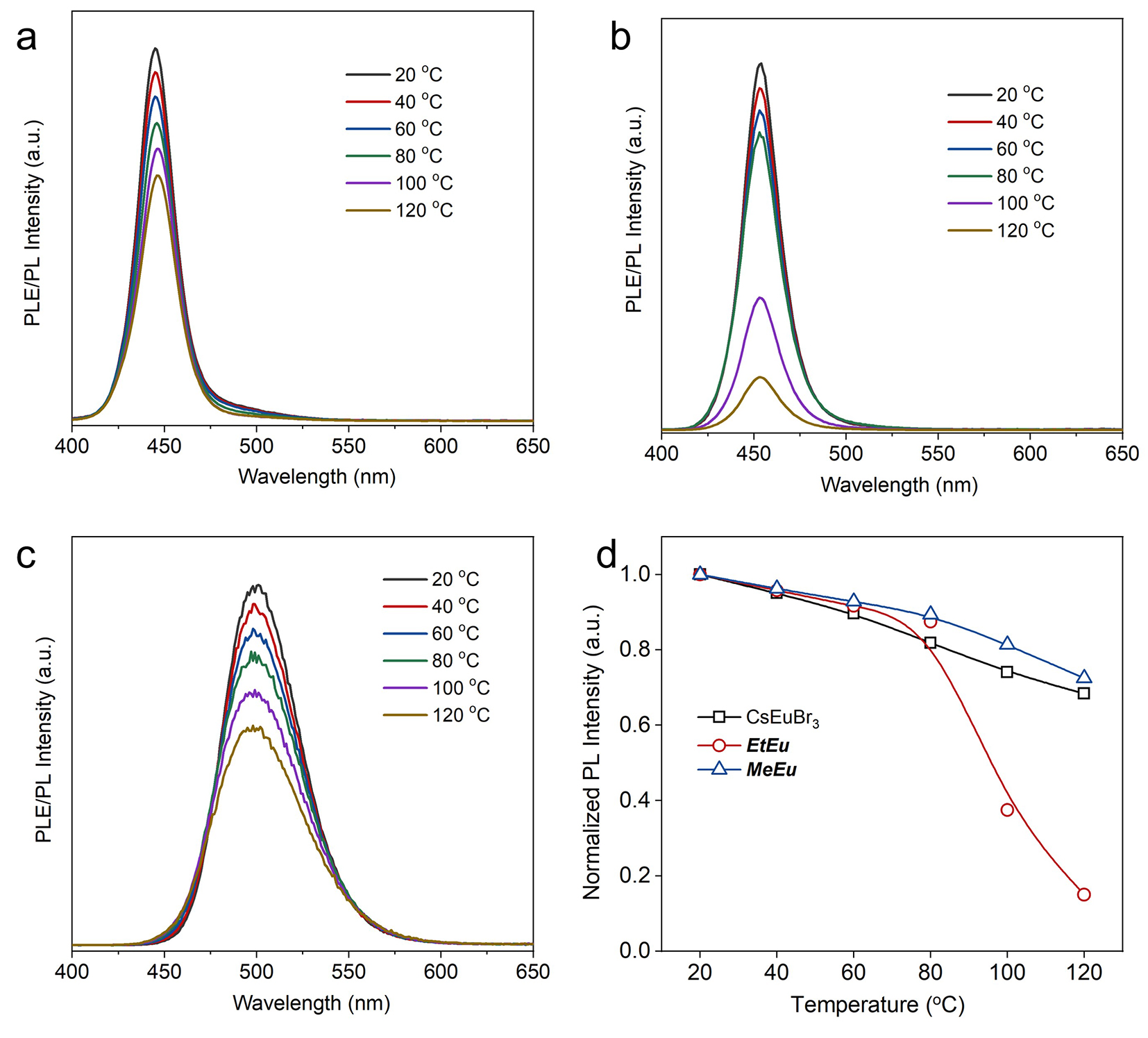


Fig. S9 The PL spectra of (a) CsEuBr_3_, (b) ***EtEu*** and (c) ***MeEu*** hybrids as a function of temperatures. (d) Fitting results of the PL intensity as a function of temperatures.

**Note:**

The thermal stability of as-prepared materials is consistent with the calculated Δ*E* value at low temperatures below 80 ^o^C. When the temperature further increases, the luminescent performance of ***EtEu*** sharply decreases, which is due to the volatilization of MeOH inside ***EtEu***. At the same time, we also noticed that the thermal stability of ***MeEu*** remained above 80% at 100 ^o^C, thanks to its higher Δ*E* value and higher structural stability (more rigid face-sharing local structure and organic cations isolated 0D structure).


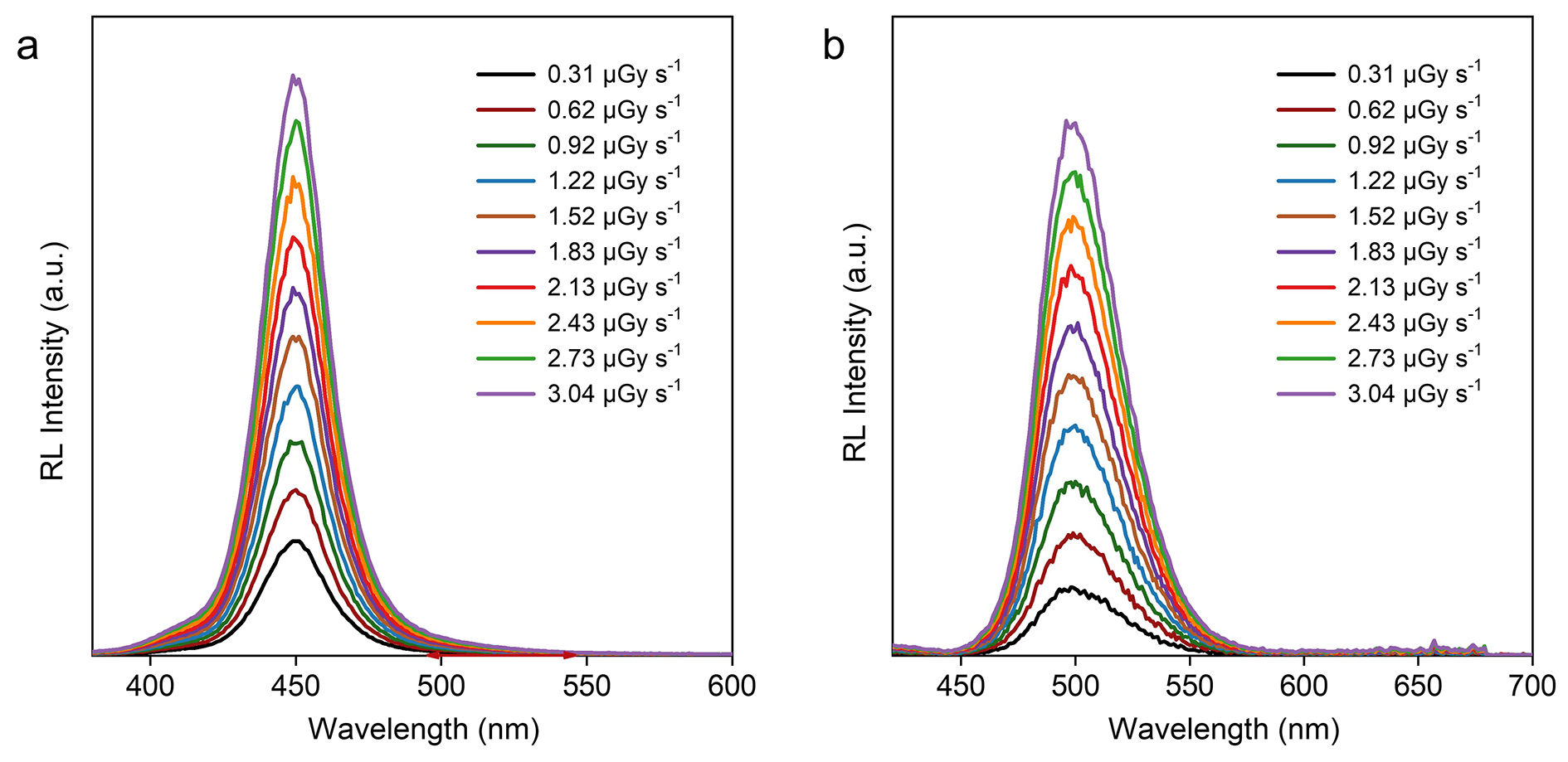


Fig. S10 **The RL spectrums dependence on X-ray dose rate.** **a** The RL spectrums of ***EtEu*** single crystals under X-ray dose rate. **b** The RL spectrums of ***MeEu*** single crystals under X-ray dose rate.


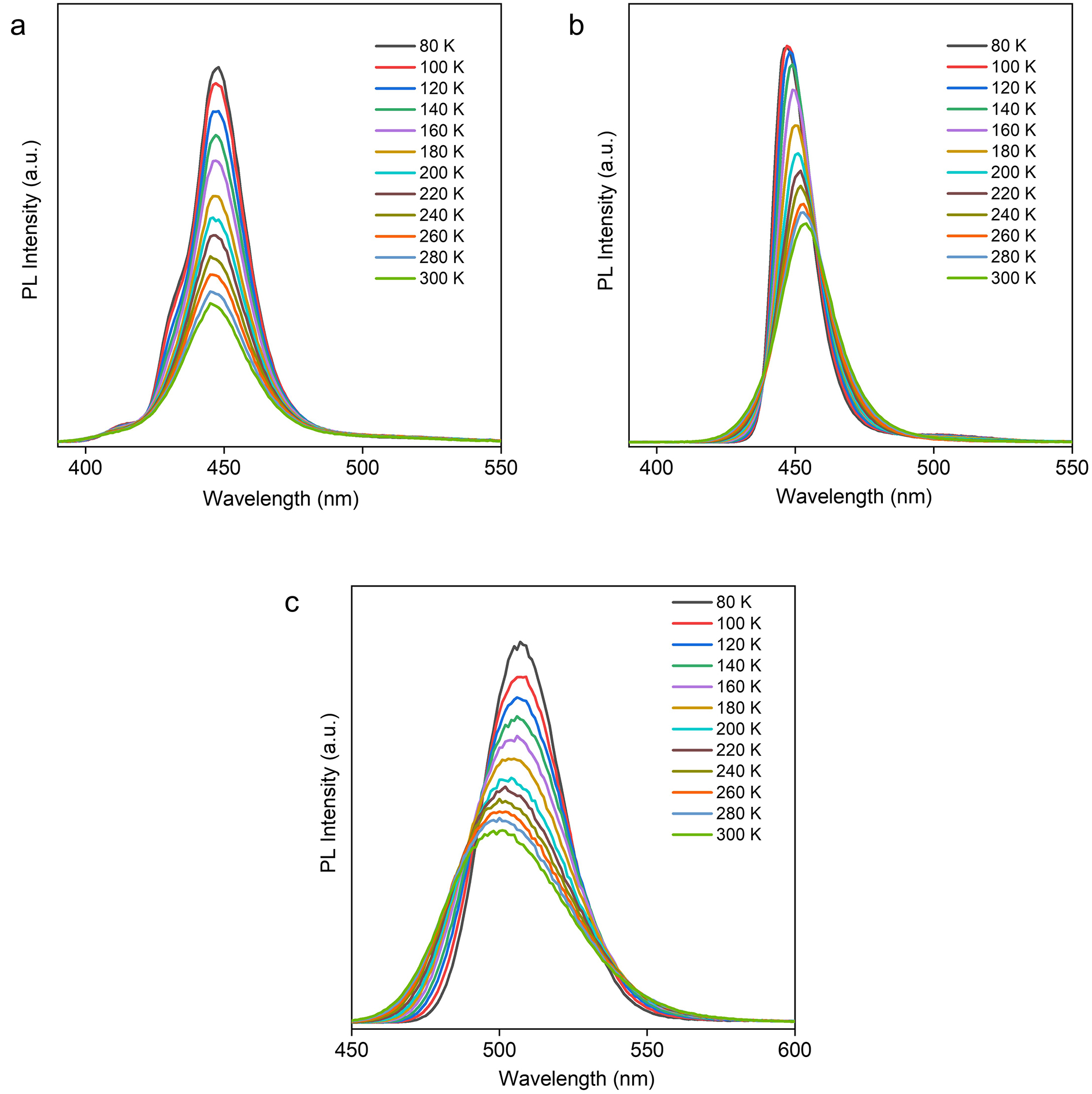


Fig. S11 **Temperature-dependent PL spectrum with temperature from 80 to 300 K.** As the temperature increases, all PL spectra of **a** CsEuBr_3_, **b** ***EtEu*** and **c *MeEu*** hybrids show a slight decrease, accompanied by the shift of peak positions, which mainly comes from the thermal vibration of the lattice.^12^


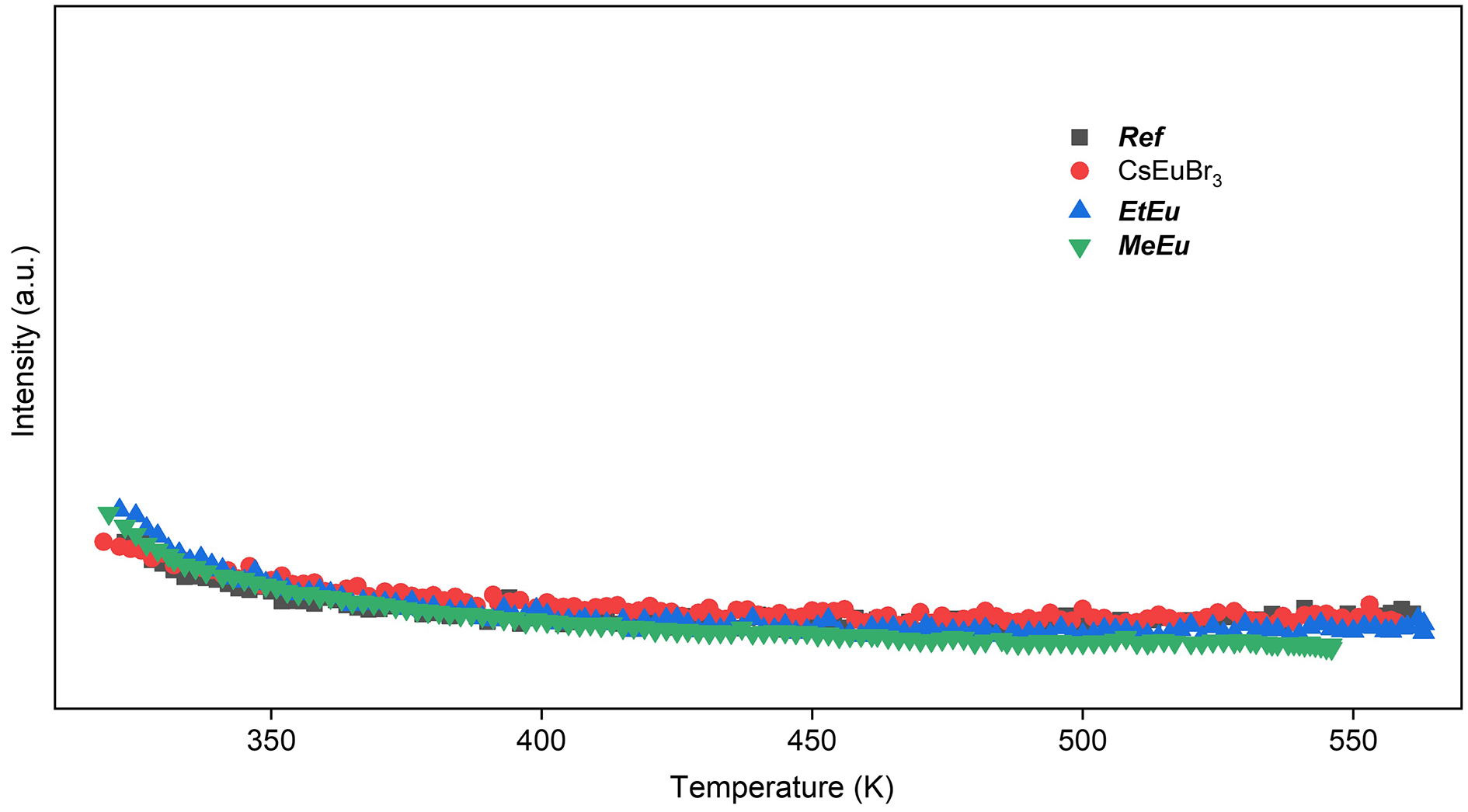


Fig. S12 Thermoluminescence curves in the temperature range 300 to 550 K after X-ray irradiation for 10 min at room temperature.


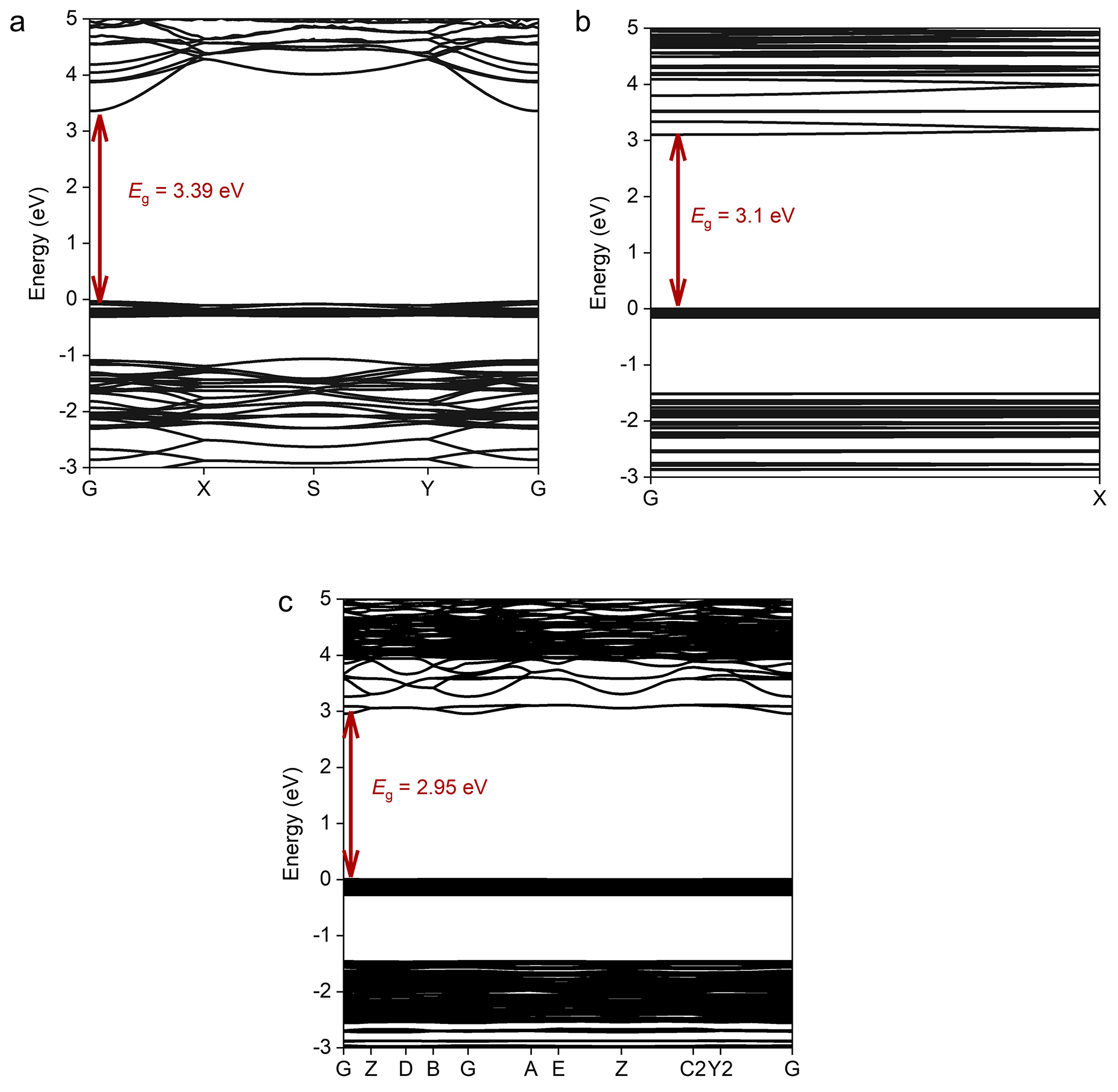


Fig. S13 Energy bandgap plots for **a** CsEuBr_3_ **b** ***EtEu*** and **c *MeEu***.

**
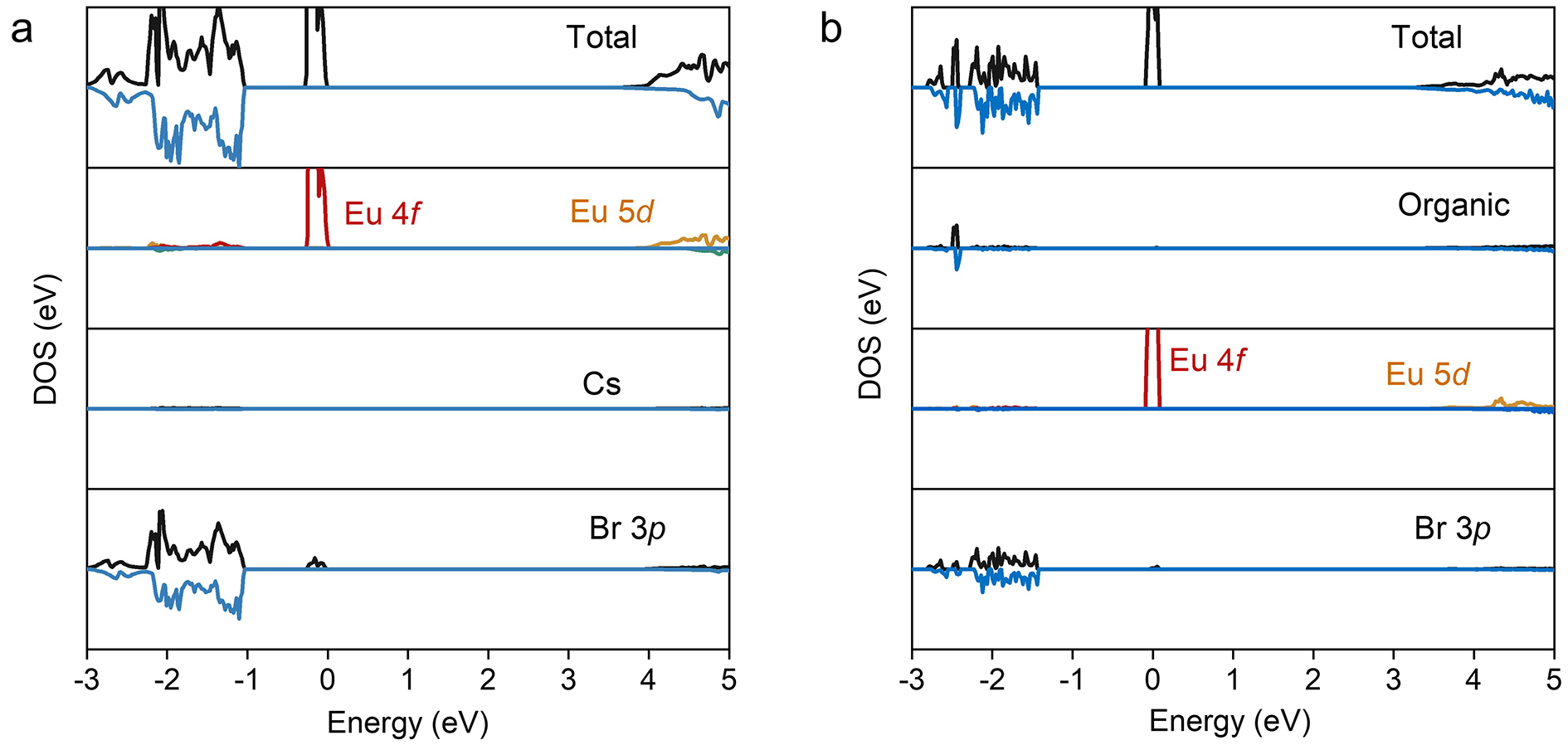
**

Fig. S14 **Electronic structures.** The density of states (DOS) **of a** CsEuBr_3_ **b** ***EtEu***.


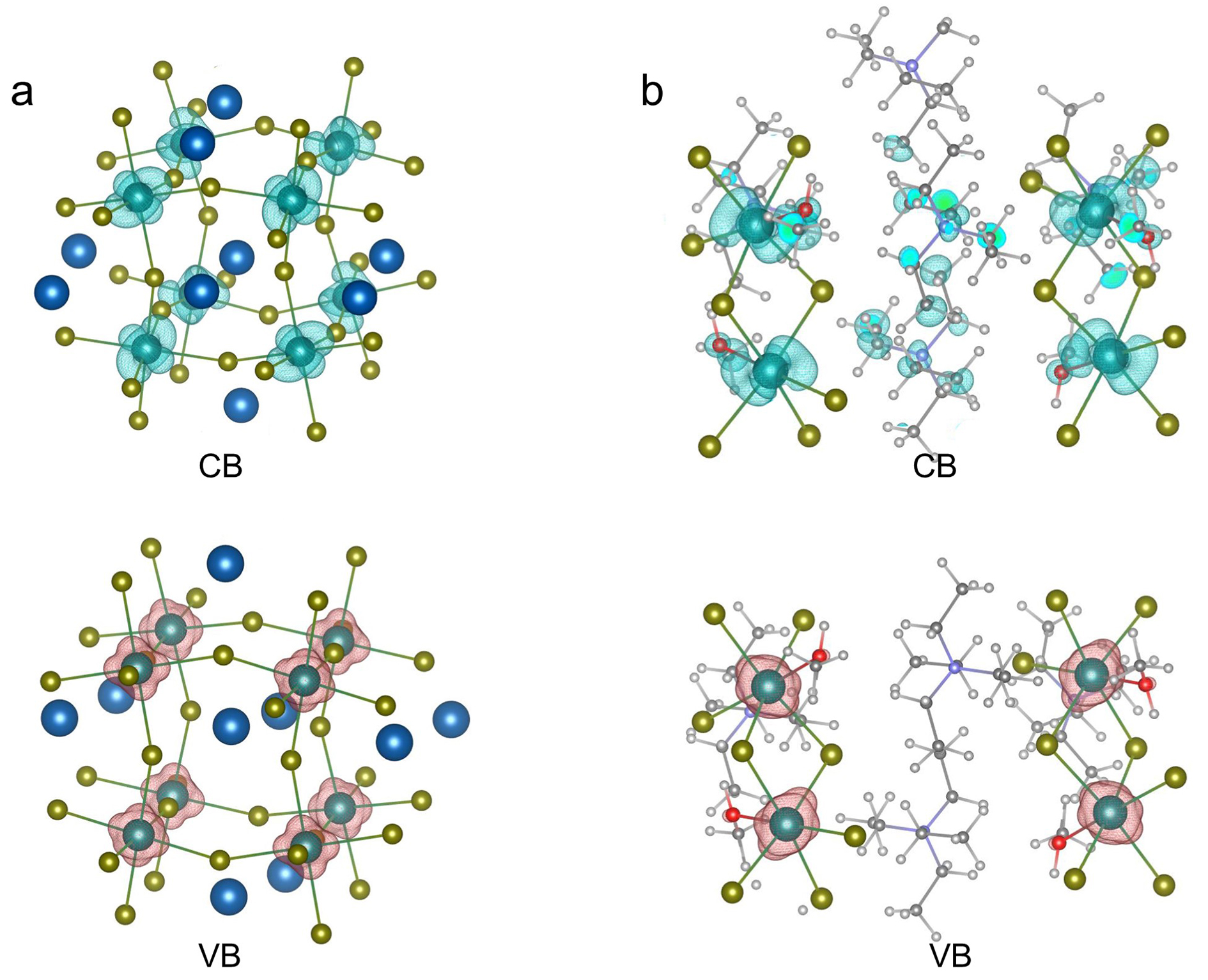


Fig. S15 **Electronic structures.** Partial density contours in the VB and CB for **a** CsEuBr_3_ **b** ***EtEu***.

**
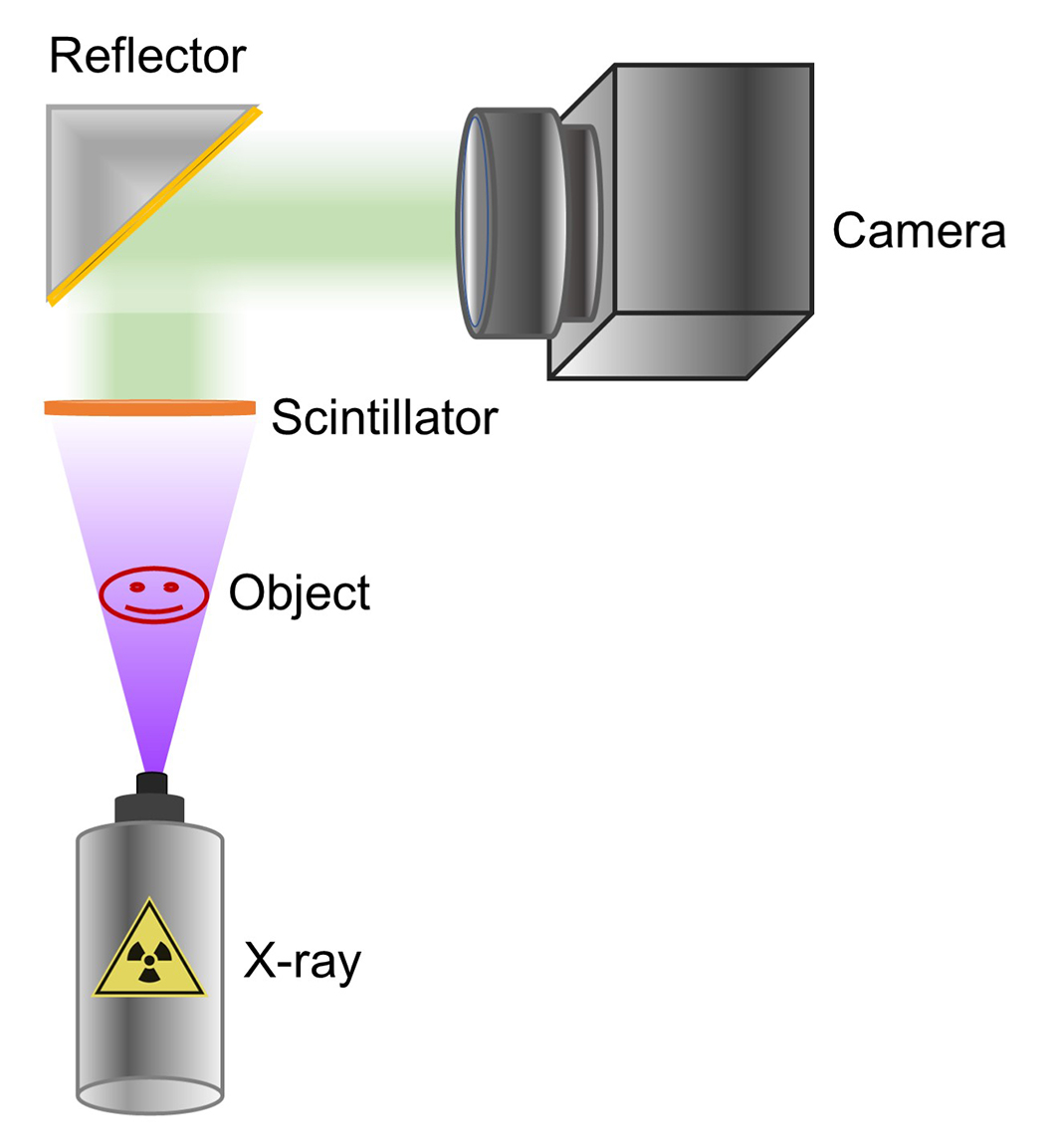
**

Fig. S16 **Schematic of the X-ray imaging system.**


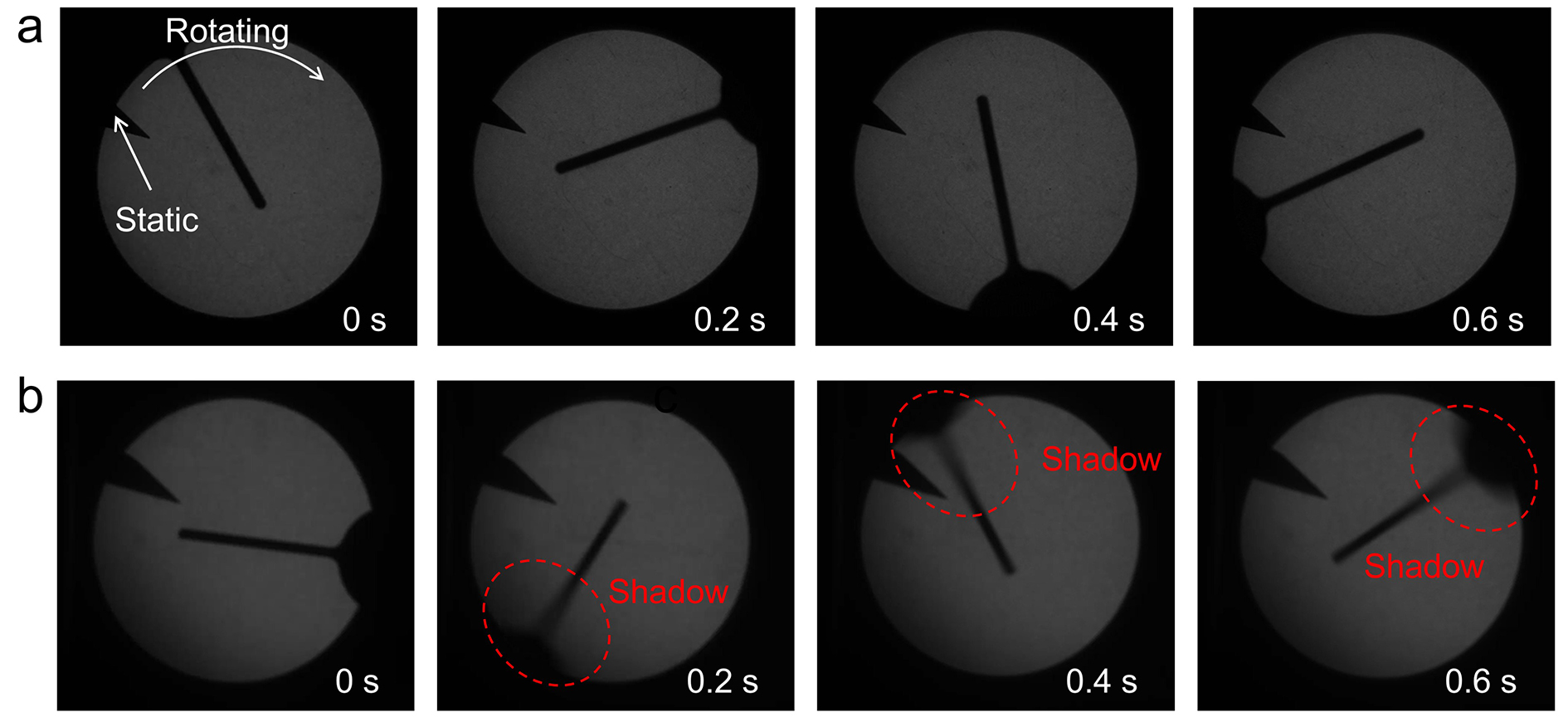


Fig. S17 Real-time dynamic X-ray imaging recording rotating and static iron needle using (a) 0D ***MeEu*** scintillator and (b) CsI:Tl scintillator.

**
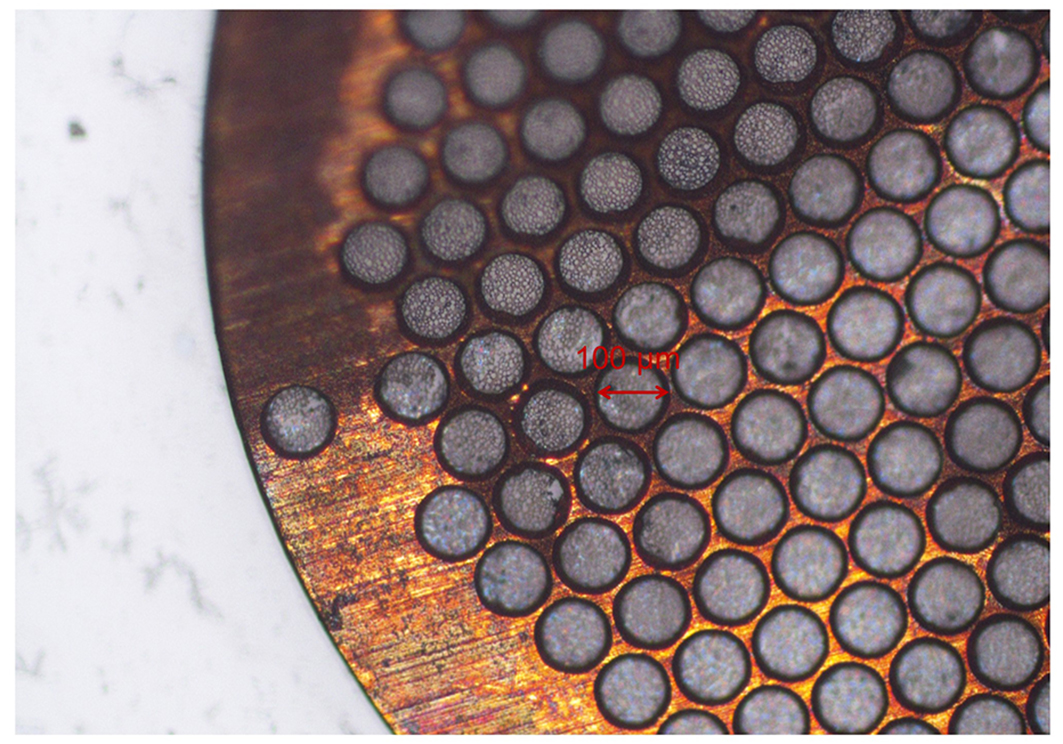
**

Fig. S18 **Photographs of copper mesh (diameter: 100 μm)**

**
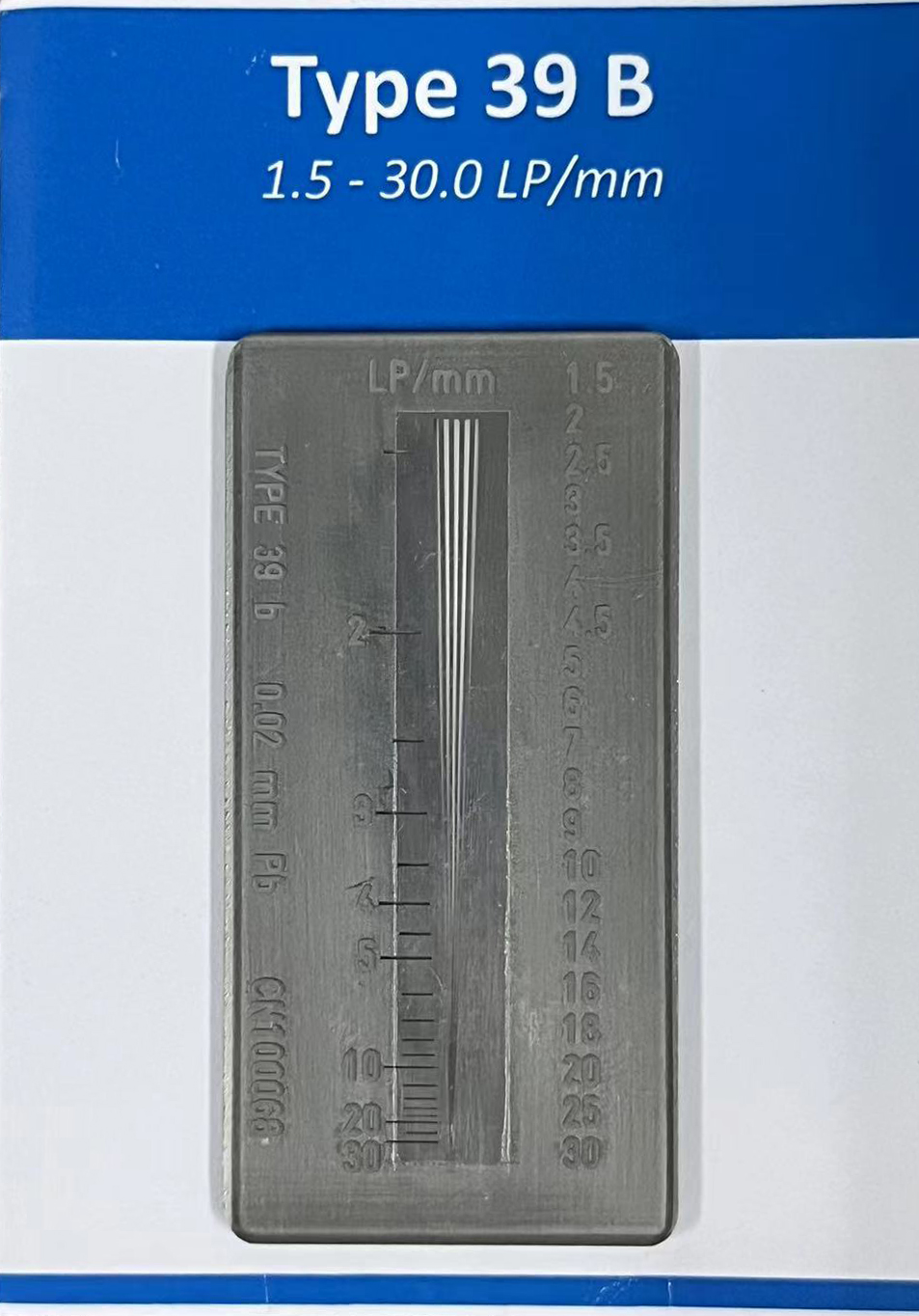
**

Fig. S19 **Photographs of a standard line-pair card (TYPE 39 b).**


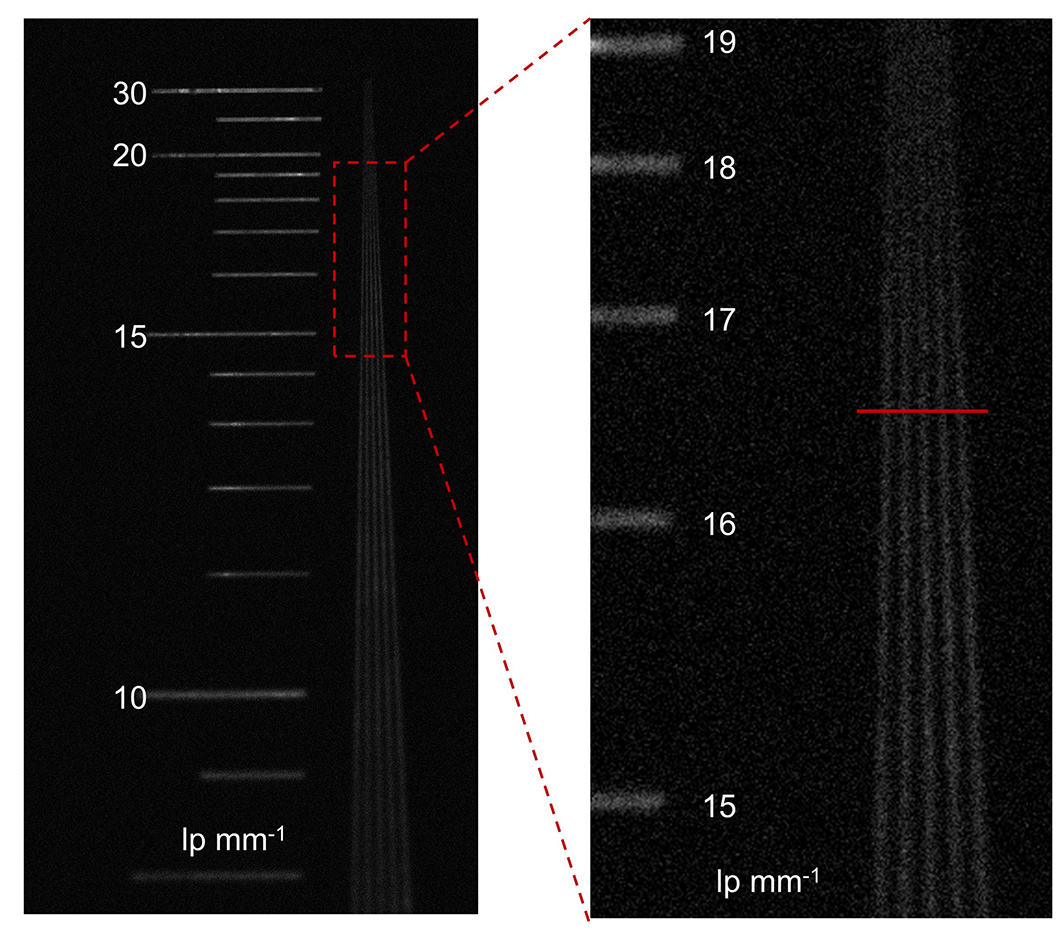


Fig. S20 **X-ray imaging of partial region of the standard x-ray test pattern based on PDMS@*MeEu*.**


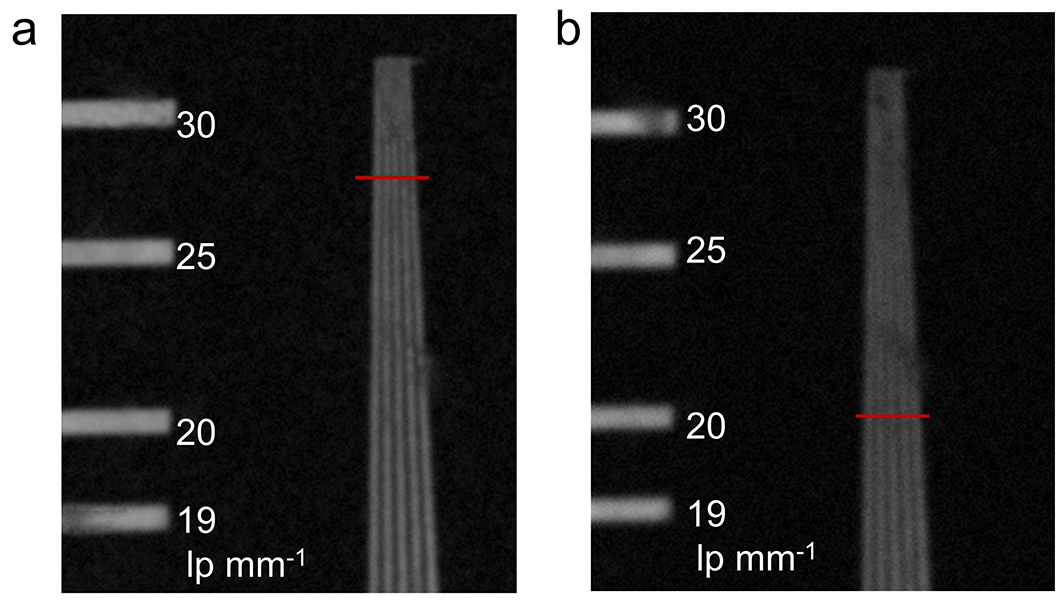


Fig. S21 X-ray imaging of partial region of the standard x-ray test pattern based on (a) AAO@***MeEu*** with glass substrates and (b) ***MeEu*** with a silica template.

**Note:**

We measured X-ray imaging using AAO thin films with high transparency glass substrates (thickness ~ 1mm) and a silica template with capillary microholes (50 μm hole diameter, 20 mm diameter, 1 mm depth, Hefei Zhuo Rui Optoelectronics Technology Co.). X-ray imaging employed AAO@***MeEu*** with glass substrates demonstrates spatial resolution up to ~ 27 lp mm^−1^, which is almost consistent with a single AAO@***MeEu*** film, superior to ~ 20 lp mm^-1^ of ***MeEu*** with a silica template. The difference in spatial resolution is mainly due to silica template with larger hole diameter (50 μm hole diameter) for AAO film (30 nm hole diameter).

# References

1. Blöchl, P. E. Projector augmented-wave method. *Phys. Rev. B* **50**, 17953-17979 (1994).

2. Perdew, J. P., Burke, K. & Ernzerhof, M. Generalized Gradient Approximation Made Simple. *Phys. Rev. Lett.* **77**, 3865-3868 (1996).

3. Anisimov, V. I., Zaanen, J. & Andersen, O. K. Band theory and Mott insulators: Hubbard U instead of Stoner I. *Phys. Rev. B* **44**, 943-954 (1991).

4. Dorenbos, P. 5d-level energies of mathrm Ce^3+^ and the crystalline environment. I. Fluoride compounds. *Phys. Rev. B* **62**, 15640-15649 (2000).

5. Dorenbos, P. Relation between Eu^2+^ and Ce^3+^ f ↔ d-transition energies in inorganic compounds. *J. Phys.: Condens. Matter* **15**, 4797 (2003).

6. Gonze, X. *et al.* Recent developments in the ABINIT software package. *Computer Physics Communications* **205**, 106-131 (2016).

7. Fang, Y.-C., Kao, P.-C., Yang, Y.-C. & Chu, S.-Y. Two-Step Synthesis of SrSi_2_O_2_N_2_: Eu^2+^ Green Oxynitride Phosphor: Electron-Phonon Coupling and Thermal Quenching Behavior. *J. Electrochem. Soc.* **158**, J246 (2011).

8. Dorenbos, P. Energy of the first 4*f*^7^→4*f*^6^5*d* transition of Eu^2+^ in inorganic compounds. *J. Lumin.* **104**, 239-260 (2003).

9. Henderson, B. & Imbusch, G. F. Optical Spectroscopy of Inorganic Solids, (Oxford University Press, 2006).

10. Nagpure, I. M. Nanoarchitectonics and properties of KMgSO_4_Cl:Eu phosphor: oxidation state of Eu ion, TL kinetic parameters and fading response. *Appl. Phys. A* **128**, 431 (2022).

11. Dorenbos, P. Thermal quenching of Eu^2+^ 5*d*–4*f* luminescence in inorganic compounds. *J. Phys.: Condens. Matter* **17**, 8103 (2005).

12. Zhao, M. *et al.* Next-Generation Narrow-Band Green-Emitting RbLi(Li_3_SiO_4_)_2_:Eu^2+^ Phosphor for Backlight Display Application. *Adv. Mater.* **30**, 1802489 (2018).
